# Insights Into the Molecular Mechanisms of Polycystic Kidney Diseases

**DOI:** 10.3389/fphys.2021.693130

**Published:** 2021-09-08

**Authors:** Valeriia Y. Vasileva, Regina F. Sultanova, Anastasia V. Sudarikova, Daria V. Ilatovskaya

**Affiliations:** ^1^Institute of Cytology, Russian Academy of Sciences, St. Petersburg, Russia; ^2^Saint-Petersburg State Chemical Pharmaceutical University, St. Petersburg, Russia; ^3^Department of Physiology, Augusta University, Augusta, GA, United States

**Keywords:** polycystic kidney disease, microbiome, mitochondria, calcium, cilia

## Abstract

Autosomal dominant (AD) and autosomal recessive (AR) polycystic kidney diseases (PKD) are severe multisystem genetic disorders characterized with formation and uncontrolled growth of fluid-filled cysts in the kidney, the spread of which eventually leads to the loss of renal function. Currently, there are no treatments for ARPKD, and tolvaptan is the only FDA-approved drug that alleviates the symptoms of ADPKD. However, tolvaptan has only a modest effect on disease progression, and its long-term use is associated with many side effects. Therefore, there is still a pressing need to better understand the fundamental mechanisms behind PKD development. This review highlights current knowledge about the fundamental aspects of PKD development (with a focus on ADPKD) including the PC1/PC2 pathways and cilia-associated mechanisms, major molecular cascades related to metabolism, mitochondrial bioenergetics, and systemic responses (hormonal status, levels of growth factors, immune system, and microbiome) that affect its progression. In addition, we discuss new information regarding non-pharmacological therapies, such as dietary restrictions, which can potentially alleviate PKD.

## Introduction

One of the most common inherited disorders in the United States is polycystic kidney disease (PKD), which is characterized by the formation of fluid-filled cysts in the kidneys. PKD has two major forms, autosomal dominant (ADPKD) and autosomal recessive (ARPKD), which are distinguished by their genetic cause, prevalence, age of onset and pattern of inheritance. The prevalence of ADPKD is much higher compared to ARPKD and is estimated to be 1 in 400 to 1 in 1,000 births (Torres et al., 2007), while the incidence of ARPKD is 1 in 26,500 live births (Alzarka et al., 2017). ADPKD is usually diagnosed later in life, while ARPKD develops during prenatal development, or in early childhood. ADPKD is mainly associated with mutations in the *PKD1* or *PKD2* genes encoding the polycystin-1 and -2 proteins (PC1 and PC2), respectively (Dong et al., 2019). The primary cause of ARPKD is mutations in the *PKHD1* gene encoding the fibrocystin/polyductin protein (FPC; Burgmaier et al., 2021).

**Figure 1:**
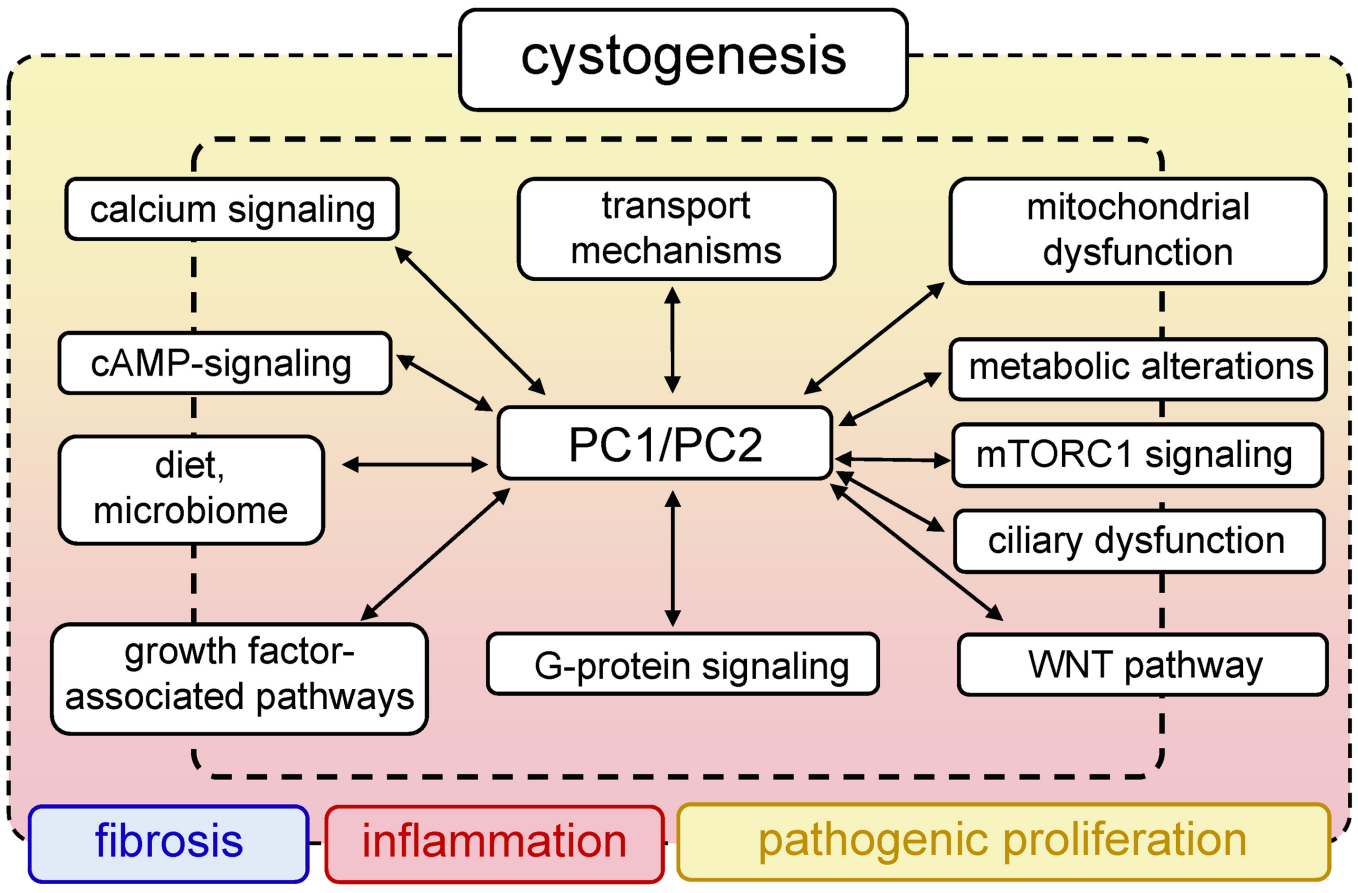
Generalized pathways involved in PKD development. Mutations in *PKD1/PKD2* lead to aberrant functionality of a variety of interconnected signaling pathways, which can result in abnormal proliferation, fibrosis, and inflammation that accompany cystogenesis.

PKD has a large list of complications that significantly impair quality of life. To date, there is no cure for PKD; however, there are drugs that can alleviate the symptoms of the disease. In 2018, we saw the first breakthrough in PKD pharmacology, when the U.S. Food and Drug Administration (FDA) approved the use of tolvaptan for ADPKD. Tolvaptan is a selective vasopressin V2 receptor antagonist that slows the progression of ADPKD, when taken in conjunction with certain dietary restrictions (Torres, 2019). However, long-term use of this drug causes serious side effects (Torres et al., 2012; Watkins et al., 2015), and there is a need to develop more effective and safe treatments. The efforts of many research teams are devoted to identification of new molecular mechanisms and potential targets in PKD. Clinical and basic science studies are underway that will reveal more information about the systemic changes associated with PKD, and shed light on the shifts in inflammatory and hormonal patterns, the microbiome, epigenetics, and other important components of this complicated pathology. The PKD research area is actively developing, and a plethora of signaling cascades and pathways have been implicated in PKD development to date. This review introduces the reader to selected classic molecular mechanisms underlying PKD, such as PC1/2 signaling, cilia-related cascades, and growth factors-related signaling. Furthermore, we will discuss some of the more recent developments in the field, such as the role of mitochondrial bioenergetics, inflammation, and implications of diet and the microbiome in PKD. Figure 1 illustrates the interconnected pathological mechanisms behind PKD, emphasizes the foundational concepts, and the complex nature of this disease. We regret that space limitations do not allow us to fully cover the vast field of PKD research. For a deeper view on PKD, we encourage the readers to refer to the latest collection of articles published in cellular signaling, in the special issue on cell signaling in PKD edited by Dr. Albert Ong and Dr. Vicente Torres (Torres and Ong, 2020), which provides a detailed insight into the recent advances in PKD research.

## Available Adpkd Treatment and the Mechanisms Behind It

Tolvaptan (Jynarque^®^), the first drug which effectively slows ADPKD progression, was approved by the FDA in 2018. Tolvaptan is a selective antagonist of vasopressin receptor 2 (V2R; Torres, 2019). In an initial phase II open-label, 3-year clinical trial among 63 patients, the administration of tolvaptan resulted in an improvement in total kidney volume (TKV) and estimated glomerular filtration rate (eGFR) in comparison with a placebo. Two pivotal clinical trials tested tolvaptan in adults with ADPKD – the 1-year REPRISE study (NCT02160145) and the 3-year TEMPO 3:4 study (NCT00428948). During the phase III, multicenter, double-blind, and placebo-controlled TEMPO 3:4 trial, tolvaptan attenuated the rate of TKV increase (2.8% vs. 5.5% in the placebo group) and slowed kidney function decline (−2.61mg/ml per year decrease in serum creatinine in tolvaptan-treated group vs. −3.81mg/ml per year in the placebo-treated group; Torres et al., 2011b, 2012; Casteleijn et al., 2017). REPRISE also showed a slower eGFR decline (−2.34ml/min per 1.73m^2^ vs. −3.61 in the placebo group) in patients with later-stage ADPKD (Torres et al., 2017b). The disadvantages of tolvaptan include moderate efficacy, significant side effects, and high cost [$5,760 per month, or $744,100 per quality-adjusted life-year gained compared with standard care (Erickson et al., 2013)]. In addition, long-term consequences of tolvaptan treatment are yet to be determined. Patients treated with tolvaptan were reported to develop side effects, such as thirst, polyuria, and liver injury. In subjects from REPRISE placebo group, percentages of individuals experiencing the levels of aspartate aminotransferase (AST) more than 3 or 20 times the upper limit of normal were 6.9 and 0.8%, while only 0.9 and 0% of patients taking tolvaptan exhibited similar AST levels (Torres et al., 2020). In TEMPO 3:4, 23.0% of tolvaptan-treated subjects exited the trial early, mostly (8.3% of subjects) due to aquaretic adverse events (Torres et al., 2012). These side effects might limit the long-term use of tolvaptan. Accordingly, basic science, clinical, and patient communities are looking forward to the discovery of new therapies in the future.

Tolvaptan slows ADPKD progression by blocking vasopressin-stimulated pathways, lowering intracellular cAMP, and inhibiting fluid secretion. In rodent models, treatment with vasopressin aggravated the development of ADPKD, while antagonism of its receptor helped preserve renal function (Hopp et al., 2015; Wang et al., 2019a). Blocking the V2R with tolvaptan can affect the extracellular signal-regulated kinase (ERK) pathway, cAMP production, and Cl^−^ secretion (Reif et al., 2011; Aihara et al., 2014; Kanhai et al., 2020). The central signaling pathways affected by tolvaptan are mediated by cAMP, which is increased at different stages in ADPKD (Cantero Mdel et al., 2015; Menezes and Germino, 2019; Sherpa et al., 2019): The elevated cAMP levels in PKD have been associated with the activation of V2R (Sherpa et al., 2019). V2R blockade reduced cAMP levels and led to a significant slowdown or prevention of cyst growth in PCK rat (ARPKD model; Wang et al., 2005). Another factor contributing to the cAMP increase in PKD is dysregulated Ca^2+^ signaling. The primary event that changes Ca^2+^ homeostasis is triggered by defects in PC1 and/or PC2 (Verschuren et al., 2018; Kuo and Chapman, 2020; Cabrita et al., 2021). Elevated levels of Ca^2+^-regulated adenylyl cyclases (AC), which synthesize cAMP, have been reported in *Pkd2*-deficient cells (Spirli et al., 2012; Wang et al., 2018). In particular, AC5 contributes to increased renal cAMP levels and cyst growth, as shown in *Pkd2* mutant mice; Wang et al. demonstrated that inhibition of AC5 may be beneficial in the treatment of PKD (Wang et al., 2018). Rees et al. showed that AC6 deficiency also can ameliorate cystogenesis, using a mouse model of PKD that was homozygous for the loxP-flanked *Pkd1* and heterozygous for an aquaporin-2-Cre recombinase transgene to achieve collecting duct-specific targeting (Rees et al., 2014).

## Current Knowledge About the Structure and Function of Pc1, Pc2, and Fpc

To date, more than 2,000 mutations in *PKD1* and about 400 mutations in *PKD2* have been identified in people with ADPKD (Dong et al., 2019). Most mutations in *PKD1* result in expression of the abnormally small, non-functional PC1 protein, which likely interrupts intracellular and ciliary signaling pathways, resulting in atypical cell growth and proliferation (Hopp et al., 2012; Cai et al., 2014; Cebotaru et al., 2014; Kurbegovic et al., 2014). Mutations in *PKD2* similarly lead to loss of PC2 function and a decrease in its expression (Kim et al., 2009; Yook et al., 2012; Douguet et al., 2019; Walker et al., 2019).

**Figure 2:**
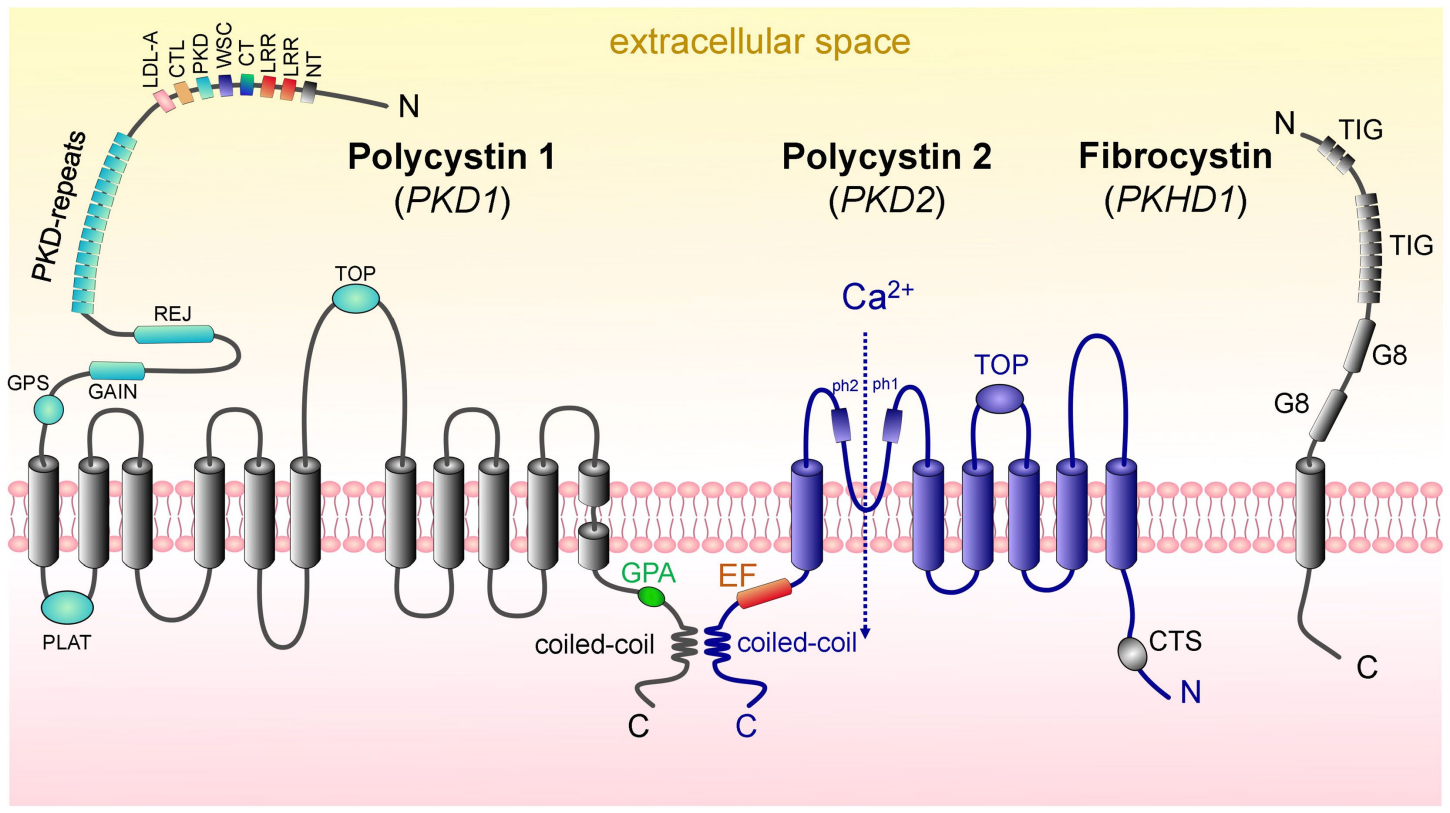
Domain structure of proteins causing ARPKD and ADPKD: polycystin 1, polycystin 2, and fibrocystin. (*left*) PC1 contains 11 transmembrane domains (TD); in addition, there are the following domains: PC1 lipoxygenase α-toxin (PLAT) domain, tetragonal opening for polycystins domain (TOP), an intracellular C-terminus with a coiled-coil domain and a G-protein activating site (GPA), an extracellular N-terminus with a G-protein site (GPS), a G protein-coupled receptors autoproteolysis-inducing (GAIN) domain, receptor for egg jelly (REJ), PKD-related repeats, low-density lipoprotein-A domain (LDL-A), C-type lectin domain (CTL), cell wall integrity and stress component domain (WSC), C-terminal cysteine-rich domain (CT), leucine-rich repeat (LRR), and N-terminal cysteine-rich domain (NT). (*middle*) PC2 has 6 TD, a tetragonal opening for polycystins domain (TOP) and two pore helices (ph1 and ph2); both of its C-and N-termini are cytosolic. Of note, its C-terminus contains an EF-hand domain and a coiled-coil domain, and in the N-tail contains a ciliary targeting sequence (CTS). PC1 and PC2 can interact *via* their coiled-coil domains. (*right*) FPC contains 1 TD, a short cytosolic C-tail, and a long extracellular N-terminus. The N-terminus contains two G8 domains, which can be involved in ligand binding and catalysis, and multiple copies of an Ig-like domain (TIG). *Abbreviations*: Polycystin 1 (PC1), polycystin 2 (PC2), transmembrane domain (TD), fibrocystin (FPC).

The PC1 protein [462-kDa, 4,303 amino acids (aa)], which is encoded by the *PKD1,* is a large integral membrane protein that has 11 transmembrane domains, a small C-terminal cytoplasmic tail and a long extracellular N-terminal tail (>3,000 aa; Hughes et al., 1995; Nims et al., 2003; Hardy and Tsiokas, 2020). PC1 is likely chemosensitive and mechanosensitive, with the overall structure similar to adhesion receptors (Figure 2; Nauli et al., 2003). One of the conserved features of PC1 is the presence of G protein-coupled receptor (GPCR) proteolytic cleavage site (GPS) and the ability of PC1 to activate G protein-mediated signaling (Parnell et al., 1998; Zhang et al., 2018). Cleavage of PC1 at its GPS is an essential step in the maturation and trafficking of these proteins (Gainullin et al., 2015). More information about these pathways in PKD and PC1’s potential function as an unconventional GPCR can be found in an excellent detailed review by Hama and Park (2016). PC2, which is encoded by *PKD2* and is also known as TRPP2, is a member of the transient receptor potential (TRP) channel family. Like all TRPs, PC2 is a non-selective Ca^2+^-permeable cation channel that is expressed in a variety of tissues. PC2 (110kDa) is a highly conserved 968 aa-long protein that contains six transmembrane domains with intracellular C-and N-terminal tails (Grieben et al., 2017). In the C-terminus, there is a coiled-coil domain that interacts with the carboxyl tail of PC1 (Figure 2; Qian et al., 1997). PC2 is known to interact with other ion channels to modulate intracellular Ca^2+^ signaling *via* the ryanodine receptor (RyR) and the inositol 1,4,5-triphosphate receptor (IP3R) in the endoplasmic reticulum (ER; Anyatonwu et al., 2007; Santoso et al., 2011). Interestingly, PC2 is required for the maturation and localization of PC1; it is hypothesized that PC2 participates in PC1 folding and quality control of this process (Gainullin et al., 2015).

PC1 and PC2 work together to help regulate cell growth and proliferation, cell migration, and cell-to-cell interactions (Nauli et al., 2003; Su et al., 2018; Nigro et al., 2019). PС1 and PС2 modulate a variety of signaling pathways. The structure, function, and stoichiometry of the PC1/PC2 complex are widely discussed (Wang et al., 2019b; Woodward and Watnick, 2019; Ha et al., 2020). PC1 and PC2 can interact *via* their coiled-coil domains, forming a heteromeric complex (likely consisting of one PC1 and three PC2 subunits; Su et al., 2018). The Ca^2+^ permeability, conductance, and functionality of the PC1/PC2 complex (or of PC2 as a homotetrameric complex without PC1) are still being debated (Kleene and Kleene, 2017; Su et al., 2018; Woodward and Watnick, 2019; Zheng et al., 2019). The association of polycystins with their partners, which can form multiprotein complexes and modulate various functions (e.g., trafficking, signaling, and degradation), is described in a comprehensive review by Hardy and Tsiokas (2020). Interestingly, recent *in vivo* studies described a potential physiologic role for PC1 in the absence of cysts, tubule dilation, or enhanced cell proliferation. The authors induced nephron-specific disruption of the *Pkd1* in 3-month-old mice and then challenged them with a high salt diet at 4–5months of age. The data suggested that PC1 deficiency in the nephron may exert a salt-wasting effect (Lakshmipathi et al., 2020). Overall, significant ongoing research efforts are devoted to the role of PC1/PC2 in ADPKD development, and not all of the existing controversies have yet been resolved. Undoubtedly, PC1/PC2 and their interaction and effects on intracellular signaling play a key role in the pathogenesis of this complex disease. We anticipate direct manipulation with the known mutations in *PKD1* through cutting-edge genetic approaches would be a very promising way to ameliorate disease progression.

The main cause of ARPKD is a mutation in the *PKHD1* gene (Outeda et al., 2017). The product of *PKHD1* is FPC, which has a length of 4,074 aa and molecular weight of 447kDa (Figure 2; Melchionda et al., 2016). The FPC is localized in the primary cilia and basal bodies in renal epithelial cells and participates in the organization of microtubules and/or mechano-and chemo-sensibilization of primary cilia, as well as in regulation of intracellular adhesion and cell proliferation (Puder et al., 2019).

## Cilia At the Forefront of the Disease: Pc1/Pc2 Complex, Ift, and Exocyst

**Figure 3:**
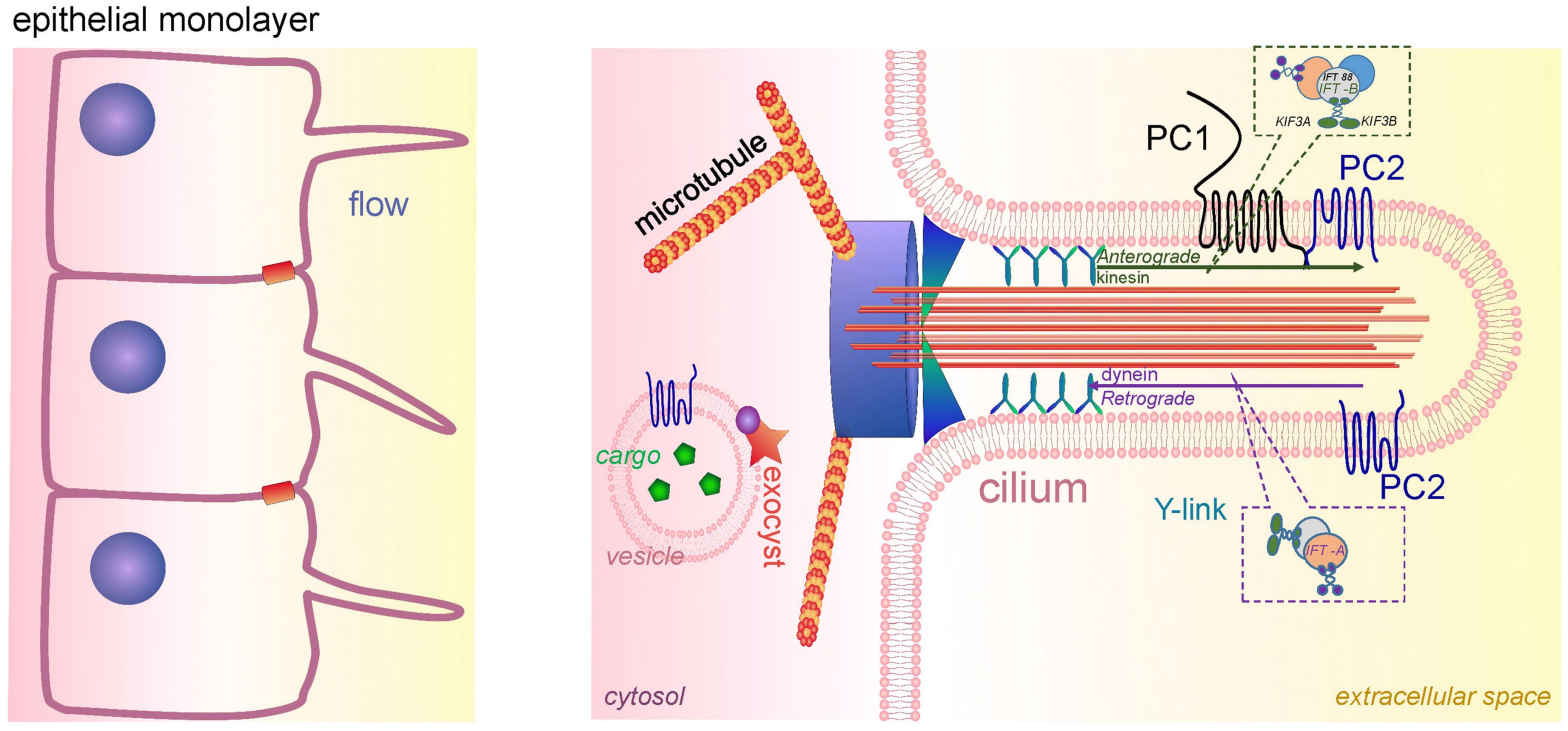
Cilia in PKD development. A schematic of the renal epithelium with cilia is shown on the left. An expanded detailed schematic of primary cilium is shown on the right. The major components of primary cilium are basal body, transition zone, axoneme, cilium membrane, and plasma. The basal body is a base of cilia that contains a lot of ciliary proteins; nine doublet microtubules extend from the basal body, forming the axoneme enclosed by the ciliary membrane and ciliary plasma. Transition zone (TZ) is a diffusion barrier which plays a critical role in ciliogenesis. TZ is formed by Y-shaped fibers, which connect axonemal microtubules to the ciliary membrane. The intraflagellar transport (IFT) system (includes anterograde and retrograde transport) is necessary for ciliary transport and cilia growth. IFT system consists of two protein complexes, IFT-A and IFT-B. The IFT-B core complex includes at least 14 IFTs, and one of the most well-studied ones is IFT88. The membrane of the cilium contains PC1/PC2 complexes, which are a part of the mechanosensitive unit of cilia. The protein complex exocyst is an important component of ciliary function; exocyst mediates the tethering of transport exocytic vesicles to the plasma membrane prior to fusion. Genetic defects in ciliary genes can cause PKD.

The primary cilium is an organelle protruding into the lumen of the renal tubular cells of different nephron segments, including proximal tubules, distal tubules, loop of Henle, and principal cells of the CD (however, primary cilia are absent on the intercalated cells; Figure 3; Liu et al., 2003). The mechanosensation of the primary cilia is debatable. According to one of the dominant hypotheses, the primary cilium acts as a unique mechano-and chemosensor that is capable of determining the presence of fluid flow as well as its composition, and regulating Ca^2+^ influx (Nauli et al., 2003; Lee et al., 2015; Pala et al., 2017). However, there is an opposite assumption that in primary cilia, mechanosensation does not regulate Ca^2+^ signaling (Delling et al., 2016). Delling and colleagues showed that the initial oscillation of Ca^2+^ occurs in the cytoplasm in response to flow and then diffuses into the cilia. However, it remains unclear what the source of the mechanically induced Ca^2+^ oscillations is in this case.

There are five main parts in the structure of the primary cilia: axoneme, ciliary membrane, ciliary plasma, basal body, and the transition zone (Figure 3). The ciliary membrane contains a plethora of receptors, ion channels, and sensory proteins, and the cilioplasma is enriched with signaling and transport proteins, which are activated in response to mechanical and chemical stimuli. The axoneme and the basal body connect through a diffusion barrier – the so-called transition zone or the gate, which is formed by the characteristic Y-shaped doublet microtubules connected to the ciliary membrane (Y-links). This barrier separates the ciliary matrix and cilioplasma from the cytoplasm. Disturbances in the structure and function of the cilia lead to a spectrum of diseases known as ciliopathies. Dysregulation of expression, maturation, and trafficking of polycystins to the cilium is shown to be involved in pathogenesis of PKD (Ma et al., 2013; Cai et al., 2014).

In the healthy kidney, the length of the renal cilia should be within a certain range, which can change during the cell cycle or as the body develops. However, significant changes in the length of the primary cilia or their loss have been linked to impaired renal function (Park, 2018). The cilia are assembled, disassembled, and maintained with the help of the intraflagellar transport (IFT) system. The most well-known mutations in IFT system genes leading to ADPKD are reported in the *IFT88*/*POLARIS* gene, resulting in loss of *IFT88*, and a mutation in the *IFT20* gene. Another gene required for the formation of the primary cilium and involved in PKD is KIF3A (Kinesin Family Member 3A, microtubule-based anterograde translocator for membranous organelles). Tissue-specific inactivation of *Kif3a* in transgenic mice leads to cystogenesis, as well as inhibition of the cilia growth in cyst-lining cells (Lin et al., 2003).

Normally, the PC1-PC2 complex in the ciliary membrane inhibits the so-called cilia-dependent cyst activation (CDCA) signal. In ADPKD, the dysfunction of PC1-PC2 promotes cyst growth due to weakening of the CDCA inhibition (Ma et al., 2013). Moreover, ciliary PC1-PC2 can regulate CDCA signals that control the diameter of renal tubular lumen or the shape of renal epithelial cells (Saburi et al., 2008; Ma et al., 2017). According to the predominant hypothesis, this complex translates the extracellular flow shear stress into a Ca^2+^ signal (Rydholm et al., 2010; Doerr et al., 2016). It should be noted, however, that some studies contradict this assumption and report complete lack of mechanically induced Ca^2+^ increases in primary cilia (Delling et al., 2016). In addition, ciliary PC1-PC2 is important for maintaining epithelial cells in differentiated and polarized states (Nauli et al., 2003; Malekshahabi et al., 2019). Inhibition of this complex leads to a decreased Ca^2+^ influx into the cell, which disrupts downstream cascades (Boehlke et al., 2010).

Another ciliary protein complex which is crucially important in PKD is the exocyst, which is a highly conserved eight-protein complex responsible for directing and securing exocytic vesicles from the trans-Golgi network to their sites of fusion with the plasma membrane (Mei et al., 2018). The exocyst is necessary for cell growth and correct development of primary cilium in renal cells (Picco et al., 2017) and includes Sec3p, Sec5p, Sec6p, Sec8p, Sec10p, Sec15p, Exo70p, and Exo84p proteins. In the primary cilia of renal tubular cells, the exocyst co-localizes and interacts with PC2 (Fogelgren et al., 2011). The Sec10 subunit is known to biochemically interact not only with PC2, but also with IFT88 and IFT20 (Fogelgren et al., 2011). Mutations in any component of the exocyst can lead to cystic kidney disease (Chacon-Heszele et al., 2014). The exocyst and its regulators are recognized as very important components of primary cilium regulation (Noh et al., 2018). Over the years, it has become clear that PKD is a ciliopathy, and manipulating ciliary proteins and signaling cascades initiated in or by cilia (especially those associated with PC1/PC2) as new targets are promising. Unfortunately, no therapies based on these mechanisms have emerged so far, and we are looking forward to the development of novel pharmacology that would allow for cilia-specific targeting.

## Hormones and Growth Factors in PKD

Based on numerous clinical and translational studies aimed at identifying novel aspects of ADPKD and ARPKD pathogenesis, growth factors have emerged as potentially important effectors. This section provides relevant information about growth factors, hormones, and cytokines that affect progression of PKD *via* effects on processes, such as cell growth, proliferation, and differentiation.

### Epidermal Growth Factor

Cellular proliferation, fibrosis, inflammation, and fluid secretion can be accelerated by the epidermal growth factor (EGF); EGF signaling is activated when EGF binds to its receptors [EGFRs, such as EGFR (also known as ERB1), ERBB2, ERBB3, and ERBB4]. EGF signaling plays a role in the expansion of renal cysts; abnormal mislocalization and overexpression of EGFR was demonstrated in human and mouse cyst-lining epithelial cells, which are also typically characterized by disturbed cell polarity. In addition, EGF and its related growth factors can directly affect sodium reabsorption in the distal nephron (Zheleznova et al., 2011). Sweeney et al. showed in 2017 that tesevatinib (TSV), a multi-kinase inhibitor (especially effective for the EGFR family), reduced phosphorylation of key mediators of cystogenesis and alleviated renal and biliary disease in bpk and PCK models of ARPKD (Sweeney et al., 2017). Taken together, the data indicate that overactive EGF/EGFR signaling contributes to the pathophysiology of AD and ARPKD, suggesting that inhibition of this pathway is a potential therapeutic target.

### Transforming Growth Factors and Fibroblast Growth Factor

In human ADPKD, the levels of transforming growth factor β1 (TGF-β1) were shown to be higher compared to the control group (Kocer et al., 2016). A recent study found that overexpression of TGF-β1 in the *Pkd1*^RC/RC^ mouse model ultimately leads to loss of renal function (Hopp et al., 2012; Zhang et al., 2020). Leonard et al. demonstrated that iKspCre-*Pkd1*^lox,lox^ mice (conditional *Pkd1* deletion leading to cystic disease) exhibit increased expression of activin ligands (members of the TGF-β family; Leonhard et al., 2016). In this study, sequestration of activin ligands was able to inhibit cystogenesis in three different mouse PKD models. Experiments performed in a 3D culture of ADPKD cells from human kidneys demonstrated the inhibitory effect of TGF-β2 on cyst formation (Elberg et al., 2012).

In another study, TGF-α was reported to be abnormally overexpressed in bpk mice (ARPKD model), potentially accelerating the disease (Dell et al., 2001). However, an increase in TGF-α is likely not the primary cause for disease progression. In a transgenic slowly progressing cystic mouse model homozygous for the *Pcy* gene, TGF-α was not required for cyst formation and did not initiate cyst development, although it was able to accelerate the enlargement of cysts once cystogenesis was initiated (Gattone et al., 1996).

Fibroblast growth factors (FGFs) are a family of cell signaling proteins that regulate various biological processes, including inflammation (Hanudel et al., 2019). Clinical studies in patients with ADPKD demonstrated higher levels of FGF23 compared to control groups. Predominantly, they observed an increase in the cleaved C-terminal fragment of FGF23, which lacks phosphaturic activity (Takenaka et al., 2020), while the FGF23 coreceptor – Klotho – was shown to be reduced in ADPKD patients and DBA/2-pcy mice (Kanai et al., 2018; Takenaka et al., 2020). Although the importance of growth factors, especially EGF, has been firmly established in AD and ARPKD, more studies are needed in order to provide more mechanistic information on the TGF and FGF23/Klotho pathway in patients with PKD, as their role still remains somewhat obscure.

### Endothelin

The endothelins (ET-1, ET-2, and ET-3) are a family of multifunctional peptides with potent effects on renal electrolyte handling, fibrosis, and inflammation (Raina et al., 2016). ET-1 has a variety of effects on renal physiology through its major receptor subtypes, ETa and ETb. In ADPKD, the circulating and local ET-1 systems are abnormally activated, and the expression of ETa and ETb receptors have been reported to be increased in human tissues (Kocyigit et al., 2019). Interestingly, the use of selective antagonists of ET-1 receptors showed that the ETa/ETb balance is critical for the progression of disease. For instance, ETb blockade accelerated cyst growth in a *Pkd2*^WS25/−^ orthologous mouse model of ADPKD (obtained by intercrossing *Pkd2*^+/−^ and *Pkd2*^WS25/WS25^ founder mice), while simultaneous blocking of ETa neutralized this effect (Chang et al., 2007). Current evidence suggests that a balance between receptor subtypes is necessary to maintain kidney structure and function, and one can hypothesize that the same is likely true for PKD.

### Renin-Angiotensin-Aldosterone System

Renin-angiotensin-aldosterone system (RAAS) is tightly linked to the high blood pressure that develops as a complication in the majority of ADPKD patients (Patch et al., 2011; Hian et al., 2016). Besides increased blood pressure, there is a possibility that dysregulation of RAAS can potentially contribute to disease progression by activating signaling cascades that promote cyst growth and especially affect epithelial transport. Kim et al. (2019) revealed that high activity of intrarenal RAAS (assessed by urinary angiotensinogen) is associated with increased excretion of K^+^ in patients with PKD, and suggested intrarenal RAAS activity as a prognostic marker for mortality and renal function decline [data obtained from the KoreaN cohort study for Outcome in patients With Chronic Kidney Disease (KNOW-CKD; Kim et al., 2019)]. However, reports regarding the effects of RAAS blockade in ADPKD are controversial. The hypertension in PKD (HALT-PKD) study demonstrated that although strict control of blood pressure in ADPKD is associated with a slower TKV increase, it is independent of the pharmacologic intensity of the RAAS blockade (Brosnahan et al., 2018). In a recent randomized control trial (NCT01853553), spironolactone (a mineralocorticoid-receptor antagonist) reduced blood pressure in ADPKD patients but did not affect the majority of markers of oxidative stress and inflammation (Nowak et al., 2019). However, despite these slightly disappointing findings, there are still unanswered questions about the role of RAAS in ADPKD. A nationwide population-based cohort study from Taiwan demonstrated that the RAAS blockade combined with statin therapy can reduce the risk of cerebrovascular events in ADPKD (Sung et al., 2017). Saigusa et al. reported that loss of cilia (*Ift88*^−/−^ mice) or PC1 (*Pkd1^−/−^* mice) is able to increase renal angiotensinogen level (Saigusa et al., 2015), and later showed that RAAS blockade attenuates renal cystogenesis in *Pkd1* mice (Saigusa et al., 2016; Fitzgibbon et al., 2018). Earlier, Loghman-Adham et al. suggested that overactivity of intrarenal RAAS results in an increased level of intratubular Angiotensin II in ADPKD patients (Loghman-Adham et al., 2004). A recent study by Salih et al. reported similar circulating RAAS component levels in ADPKD and chronic kidney disease (CKD) patients; however, higher urinary excretion of angiotensinogen and renin was found to be a unique feature of ADPKD (Salih et al., 2017). Konvalinka et al. reported lower urinary excretion of Angiotensin II-regulated proteins (BST1, LAMB2, LYPA1, RHOB, and TSP1) in ADPKD compared to CKD, which was attributed to a lack of communication between cysts and tubules (Konvalinka et al., 2016). It should be emphasized that hypertension is a complication of ADPKD-induced chronic kidney failure and cannot be regarded as a direct cause of disease. Alleviating high blood pressure can indeed help the prognosis of ADPKD treatment, but the treatment of hypertension should not be equated with the treatment of ADPKD.

In ARPKD, the data related to RAAS are rather limited. A study in a PCK rat model reported that the intrarenal, but not systemic, RAAS activation was associated with ARPKD (Goto et al., 2010). In earlier studies, conflicting data were reported, showing both increased and decreased RAAS components in ARPKD patients and the Lewis rat, an ARPKD model (Loghman-Adham et al., 2005; Phillips et al., 2007). More controlled, large-scale studies are required to improve our understanding of the role of RAAS blockade in the management of hypertension during PKD, potential involvement of RAAS in the molecular mechanisms underlying AD and ARPKD pathophysiology, and the usability of its components as prognostic biomarkers for PKD progression.

## Mitochondria-Related Signaling Pathways

**Figure 4:**
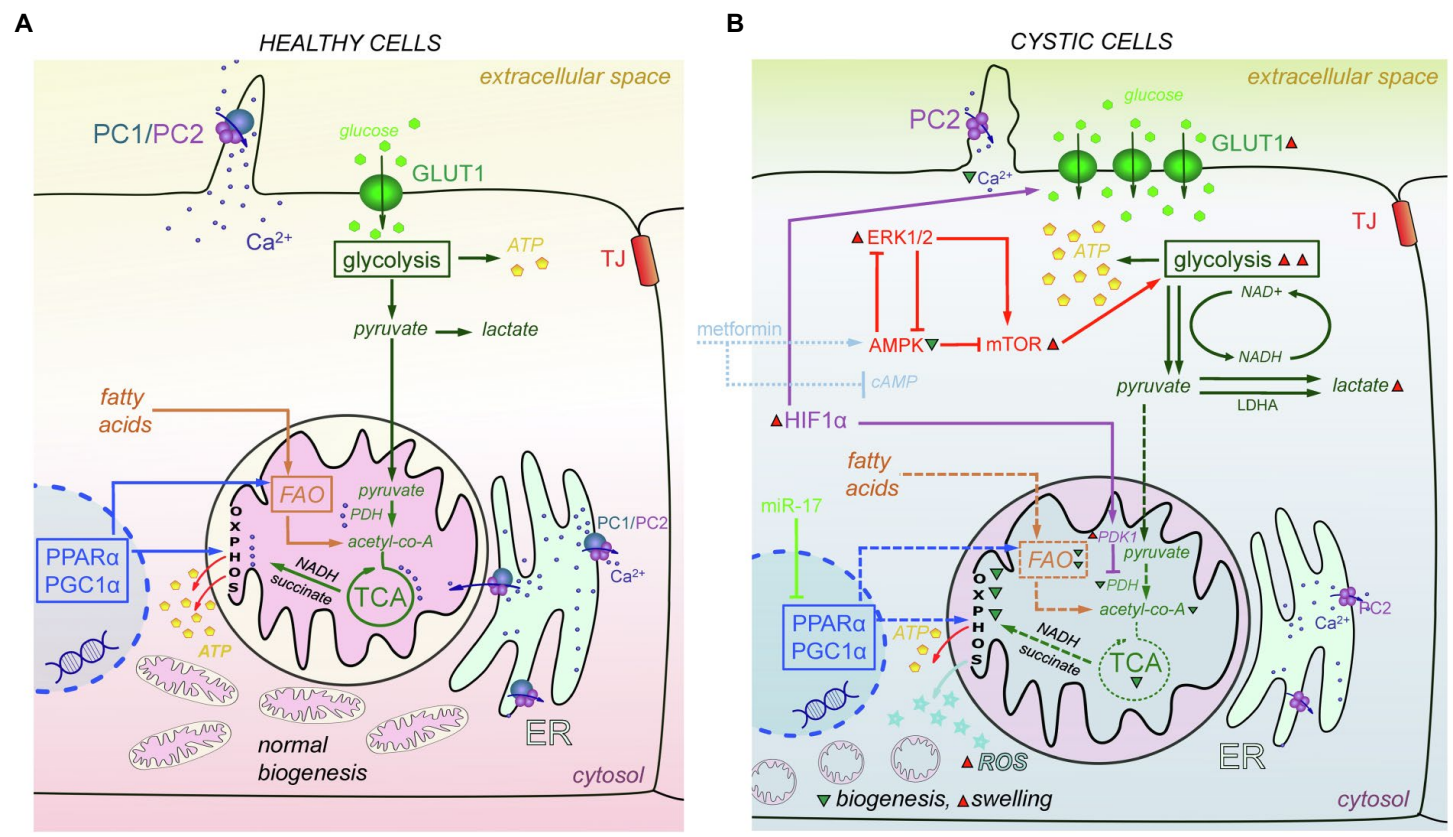
Metabolic and mitochondria-mediated effects in PKD. **(A)** In mitochondria of the healthy cells, PDH converts pyruvate to acetyl-CoA. Acetyl-CoA is the main fuel for the TCA cycle, which produces NADH and succinate needed for OXPHOS to create ATP molecules. PPARα and PGC1α promote energy metabolism by affecting FAO and OXPHOS and help maintain normal mitochondrial biogenesis. PC1/PC2 complexes in cilia and mitochondria-associated membranes of the ER keep Ca^2+^ concentration level in range that is sufficient to successfully modulate mitochondrial metabolism, facilitate the work of TCA cycle enzymes, and affect ATP synthase activity. **(B)** In cystic cells, there is a Warburg shift in ATP production from OXPHOS to glycolysis. Overexpression of HIF-1α increases the expression of GLUT1, which allows more glucose molecules to pass into the cell. In addition, HIF-1α affects PDK1 that inhibits PDH and reduces conversion of pyruvate to acetyl-CoA. miR-17 inhibits PPARα and PGC1α, which decreases OXPHOS, FAO, and mitochondria biogenesis, and results in ROS production and mitochondrial swelling. In addition, the abnormalities of Ca^2+^ transport caused by PC1/PC2 dysfunction also decrease OXPHOS and TCA cycle efficacy, and PDH activity. The AMPK-ERK-mTOR pathway can be targeted to curtail mitochondrial metabolic abnormalities; metformin and other similar drugs can activate AMPK and (independently) suppress cAMP production, which also affects mitochondria. ERK1/2 inhibits AMPK activity and upregulates mTORC1, which then stimulates aerobic glycolysis, increases ATP production, and further inhibits AMPK. *Abbreviations:* pyruvate dehydrogenase (PDH), oxidative phosphorylation (OXPHOS), affecting fatty acid oxidation (FAO), AMP-activated protein Kinase (AMPK), extracellular signal-regulated kinase (ERK), mammalian target of rapamycin complex 1 (mTORC1), peroxisome proliferator-activated receptor-α (PPARα), peroxisome proliferator-activated receptor γ coactivator 1α (PGC1α), reactive oxygen species (ROS), pyruvate dehydrogenase kinase 1 (PDK1), hypoxia-inducible transcription factor-1α (HIF-1α). Green triangles denote a decrease in substance/protein, while red triangles denote an increase.

Mitochondria, the cellular powerhouses and important signaling nodes, can be linked to virtually every signaling pathway, and mitochondrial abnormalities have recently been directly implicated in cystogenesis. Oxidative stress is often observed in cystic cells (Ishimoto et al., 2017; Nowak et al., 2018; Andries et al., 2019). Structural damage has been reported in mitochondria in the cystic cells, which are more swollen, and exhibit indistinct and damaged cristae (Ishimoto et al., 2017). Cyst-lining cells from heterozygous Han:SPRD Cy (Cy/+) rats and Ksp-Cre *Pkd1*^flox/flox^ mice were reported to have increased ROS production and reduced mitochondrial DNA (mtDNA) copy numbers compared with their wild-type counterparts (Ishimoto et al., 2017). It is established that metabolic reprogramming occurs in cystic cells as a result of their need for intensive proliferation and growth (Podrini et al., 2020). Figure 4 summarizes the mechanisms of metabolic changes described below.

In healthy cells, glucose metabolism includes glycolysis that converts glucose to pyruvate. Pyruvate is transported to mitochondria, where it is converted to acetyl-CoA, which in turn goes through the tricarboxylic acid cycle (TCA) cycle to produce the NADH and succinate that are needed to create energy in oxidative phosphorylation (OXPHOS). However, in cystic cells, pyruvate is more intensely converted to lactate (Beck Gooz et al., 2014; Riwanto et al., 2016). This process is known as a shift from OXPHOS to aerobic glycolysis or the “Warburg effect”; a similar shift from OXPHOS to glycolysis was discovered in *Pkd1−/−* mouse embryonic fibroblasts (MEFs), kidneys from humans with ADPKD (Rowe et al., 2013), and murine models of ADPKD (Rowe et al., 2013; Chiaravalli et al., 2016; Riwanto et al., 2016). 2-deoxy-D-Glucose (2-DG, glucose analog that cannot be metabolized) reduced cyst growth in murine models of ADPKD (Rowe et al., 2013; Chiaravalli et al., 2016; Nikonova et al., 2018), heterozygous Han:SPRD rats (Cy/+; Riwanto et al., 2016), and ADPKD miniature pigs (Lian et al., 2019). Likely due to increased glycolysis, glycosphingolipid (GSL) accumulation was observed in PKD, for instance, in *Pkd1* conditional knockout mice (Natoli et al., 2010; Rogers et al., 2016) and PCK rats (Ruh et al., 2013). GSLs play a role in the formation and function of the cilia, but they can also prompt mitochondrial permeability transition and outer membrane permeability, which leads to increased ROS release and oxidative stress. Some studies showed increased expression of the key glycolytic genes, hexokinase 1 (*HK1*) and *HK2*, in human ADPKD primary cells (Chen et al., 2015; Riwanto et al., 2016). Furthermore, the cellular promoter of the Warburg effect, hypoxia-inducible transcription factor-1α (HIF-1α), and its target gene pyruvate dehydrogenase kinase 1 (*PDK1*), which keeps pyruvate away from the TCA cycle, were upregulated in *Pkd1^−/−^* cells. HIF-1a plays a protective role *via* HIF-1α-mediated mitophagy in acute kidney injury. Renal tubular-specific HIF-1α knockout mice exhibit decreased mitophagy, increased apoptosis, and elevated ROS production induced by hypoxia/reoxygenation (Fu et al., 2020). HIF-1α also regulates the expression of glucose transporter 1 (GLUT1) and increases glucose permeability in cystic cells (Rowe et al., 2013).

The metabolic alterations observed in PKD may be mediated by the ERK pathway. ERK1/2 inhibits AMPK (AMP-activated protein kinase) activity. On the other hand, ERK1/2 can upregulate mTORC1 (mammalian target of rapamycin complex), which then stimulates aerobic glycolysis, increases ATP production, and further inhibits AMPK. Upregulation of the AMPK pathway can downregulate ERKs activity toward the basal level (Rowe et al., 2013). There is evidence to suggest that stimulation of the AMPK pathway might be a promising means for new ADPKD treatments (Takiar et al., 2011; Chang et al., 2018). Recently, AMPK has been shown to phosphorylate and inhibit cystic fibrosis transmembrane conductance regulator (CFTR), an ion channel mediating renal Cl^−^ secretion. Interestingly, caloric restriction also reduced cystogenesis in ADPKD models (*Pkd1*^RC/RC^ and *Pkd2*^WS25/−^ mice), by downregulating the AMPK and the mammalian target of rapamycin (mTOR) pathway (Warner et al., 2016). Metformin, an activator of AMPK, was able to slow cystogenesis by inhibiting both the CFTR and mTOR *in vitro*; however, metformin can work independently of AMPK, by inhibiting AC and suppressing cAMP production (Takiar et al., 2011; Rees et al., 2014). Currently, a large clinical trial (Therapeutic Administration of MEtformin-PKD; NCT02656017) is probing whether stimulation of AMPK (using metformin) can be utilized as an effective and safe therapy in ADPKD (Figure 4).

Dietary interventions, such as fasting and ketogenic diets, which downregulate aerobic glycolysis because of low glucose availability, ameliorated cyst growth in an orthologous mouse model of ADPKD with a mosaic conditional knockout of *Pkd1* (Kipp et al., 2016), in *Pkd1*^RC/RC^ and *Pkd2*^WS25/-^ mice (Warner et al., 2016), and in rat (Han:SPRD Cy/+), mouse (*Pkd1*^cond/cond^:*Nes*^cre^), and feline models of PKD (Torres et al., 2019). However, the dependence of cystic cells on aerobic glycolysis is controversial. For instance, although Menezes et al. observed reduced OXPHOS in *Pkd1^−/−^* cells, they also reported that these cells used fatty acids as their primary energy source (Menezes et al., 2016). Overall, there is consensus regarding the OXPHOS reduction, among other metabolic changes in ADPKD (Menezes et al., 2016; Padovano et al., 2017; Lakhia et al., 2018; Podrini et al., 2018). Rowe et al. discovered a weak contribution of OXPHOS to ATP production in *Pkd1^−/−^* MEFs (Rowe et al., 2013). Several other studies also reported a reduced oxygen consumption rate in *Pkd1^−/−^* mouse cortical collecting duct cell line (Menezes et al., 2016), *Pkd1^−/−^* mouse renal proximal tubule cell lines (Padovano et al., 2017), and *PKD1^−/−^* human tubular cells (Ishimoto et al., 2017).

Reduction of OXPHOS occurring in PKD may be associated with decreased expression of peroxisome proliferator-activated receptor γ coactivator 1α (PGC1α), a master regulator of mitochondrial biogenesis. Interestingly, PGC1α is activated by AMPK and affects a major transcriptional factor, PPARα (Jager et al., 2007). Both PGC1α and PPARα promote energy metabolism by affecting fatty acid oxidation (FAO) and OXPHOS (Figure 4; Hajarnis et al., 2017; Ishimoto et al., 2017; Lakhia et al., 2018). In human and mouse renal cysts, the whole network of PPARα-target genes was shown to be downregulated (Song et al., 2009; Hajarnis et al., 2017), while increasing *Pparα* expression was able to attenuate cyst growth (Hajarnis et al., 2017). In a *Pkd1*^RC/RC^ mouse model of ADPKD, treatment with PPARα agonist fenofibrate increased mitochondrial function as well as slowed cyst growth and the proliferation of cystic epithelial cells (Lakhia et al., 2018).

There is evidence that the polycystin complex (PC1/PC2) by itself affects cellular energy metabolism. It was shown that both PC1 and PC2 are localized at the mitochondria-associated ER membranes (MAMs; Padovano et al., 2017). PC1/PC2 forms Ca^2+^-permeable ion channels and can modulate mitochondrial enzymes, such as pyruvate dehydrogenase phosphatase (PDP), isocitrate dehydrogenase (IDH), and oxoglutarate dehydrogenase (OGDH), that are important in TCA cycle and also affect ATP synthase activity (Rossi et al., 2019). Interestingly, cells lacking PC1 have decreased Ca^2+^-dependent OXPHOS activity (Figure 4; Padovano et al., 2017). In addition, the reduction of PC2 expression affects mitochondria Ca^2+^ buffering and increases the fragmentation of the mitochondrial network (Kuo et al., 2019). Clearly, the dysregulation of mitochondrial metabolism is an emerging and intriguing area of interest in PKD research, and there are many pathways under consideration that might lead to the development of novel treatments. We believe that mitochondrial bioenergetics are one of the most exciting targets in PKD and are looking forward to studies uncovering the intertwined network of mitochondria-mediated signaling cascades involved in cystogenesis.

## Dietary Interventions in Pkd

A growing number of studies report that PKD progression can be mitigated using non-pharmaceutical strategies, such as dietary interventions. For instance, water, salt, phosphate, and protein intake can potentially affect cyst growth (Torres et al., 2011a; Omede et al., 2020). Furthermore, caloric restriction (Kipp et al., 2016; Warner et al., 2016), 2-DG intake (Chiaravalli et al., 2016), and ketogenic diet (Torres et al., 2019) proved effective in slowing down ADPKD progression in animal models.

### Salt Intake

Salt intake is crucial for CKD. Two recent large-scale projects, the Consortium for Radiologic Imaging Studies of Polycystic Kidney Disease (CRISP) and HALT-PKD, both showed a correlation between increased salt intake and severity of ADPKD progression. CRISP demonstrated that higher urinary sodium excretion (a surrogate of salt intake) was associated with greater GFR decline and increased TKV, which are indicative of functional and structural disease progression (Torres et al., 2011a). HALT-PKD, a randomized clinical trial, investigated if strict blood pressure control affects ADPKD progression (Torres et al., 2014). Sodium restriction in HALT-PKD resulted in a modest reduction in urinary sodium excretion (Torres et al., 2017a). Both HALT-PKD and CRISP showed that salt restriction is beneficial to the management of ADPKD, although in a PCK rat (an ARPKD model) an extreme restriction of dietary salt promoted cyst growth (Ilatovskaya et al., 2019). There is a U-shaped rather than a linear relationship between sodium intake and cardiovascular outcomes (Heerspink and Ritz, 2012; Middleton and Lehrich, 2014). For instance, in patients without renal disease not only higher sodium excretion, but also lower sodium excretion as well is associated with an increase in cardiovascular death (O’Donnell et al., 2011). Therefore, the non-linear relationship between salt intake and cardiovascular outcomes should be taken into consideration when designing and interpreting studies, and recommending low salt/salt-deficient diet to patients.

### Water Intake

Apart from salt, water intake is another dietary factor to be considered in PKD. Increased water intake in PCK rats suppressed renal effects of vasopressin, which led to decreasing intracellular cAMP levels (Nagao et al., 2006). Preclinical evidence provided recently showed that an increase in water consumption leads to a reduction in ADPKD progression in the LPK (Lewis polycystic kidney) rat model (Sagar et al., 2019). However, the clinical effectiveness of higher water intake is still an open question, and interventional studies are warranted. There are several recently completed or ongoing randomized clinical trials that explored if increased water intake ameliorates ADPKD progression. One of them is a PREVENT-ADPKD pilot trial [ACTRN12614001216606 (Wong et al., 2018)], which should be completed in 2021, and another study is “Determining feasibility of Randomisation to high vs. *ad libitum* water INtake in polycystic Kidney disease” [DRINK; NCT02933268 (El-Damanawi et al., 2018)]. The DRINK trial has already been completed, and adult ADPKD patients with high water intake (HWI) during 8weeks showed lower urine osmolality and higher urine volume compared with patients with *ad libitum* water intake. HWI did not result in acute effects on GFR (El-Damanawi et al., 2020).

### Caloric/Protein Restriction and Acid Precursors Intake

Three independent papers showed that caloric and protein restriction can decrease renal cyst growth during ADPKD (Kipp et al., 2016; Warner et al., 2016). It is known that ADPKD cysts have altered glucose metabolism, since there is a Warburg-like shift (for more on this, see in the section of this review featuring mitochondria-related mechanisms in PKD). The discovery of increased aerobic glycolysis in cysts allowed to suggest the use 2-DG to attempt to curtail disease development. It was proven that inhibition of the pyruvate to lactate conversion pathway by 2-DG can slow the disease progression in *Ksp-Cre:Pkd1*^*flox/*−^ mouse model and a Han:SPRD rats (Cy/+) rat model of ADPKD (Rowe et al., 2013; Riwanto et al., 2016). Reduced food intake also decreased serum glucose level and ameliorated ADPKD progression in *PKD^cond/cond^: Nes^Cre^* (Kipp et al., 2016) and *Pkd1^RC/RC^* (Warner et al., 2016) mouse models, and the mechanisms were, respectively, reported to be mediated by activation of the AMPK pathway, which is a negative regulator of mTOR (Rowe et al., 2013; Kipp et al., 2016; Riwanto et al., 2016; Warner et al., 2016). Interestingly, even a very moderate caloric restriction (−23%) significantly decreased cyst growth and reduced interstitial fibrosis and proliferation of cystic epithelia (Kipp et al., 2016). It should be noted, however, that using restricted food intake is not easy for patients to adhere to over the long term. Along these lines, an alternative to caloric restriction, time-restricted feeding (TRF), also slows cyst growth and even protects animals from the negative effects of fasting (Torres et al., 2019). During TRF experiments, Torres et al. noticed increased levels of β-hydroxybutyrate (BHB), which is a marker of ketosis. They showed that a ketogenic diet (another way to inhibit aerobic glycolysis) lowers glucose levels and slows PKD progression in juvenile and adult Han:SPRD rats, a model of ADPKD. In addition, BHB can replace succinyl-CoA in the TCA cycle and improve mitochondrial efficiency (Torres et al., 2019). The Ketogenic Dietary Interventions in Autosomal Dominant PKD (Keto-ADPKD) clinical trial started at the end of 2020 (NCT04680780, https://clinicaltrials.gov/ct2/show/NCT04680780).

Many studies in CKD models show that keeping hydrogen ions levels low in plasma and urine is beneficial for disease mitigation (Tanner and Tanner, 2000; Mahajan et al., 2010), while increased acid intake during CKD causes rapid renal failure (Banerjee et al., 2015) and decreases GFR (Goraya et al., 2012). It is still unclear specifically how hydrogen ions facilitate cyst growth; however, increased proton excretion was correlated with decreased ADPKD progression, while administration of alkali was able to reverse that trend (Cowley et al., 1996). The clinical effectiveness of this dietary approach is yet to be explored.

Several studies highlighted that animal protein intake can affect early kidney failure during ADPKD (Klahr et al., 1995; Ogborn and Sareen, 1995). A very interesting trial conducted from 2012 to 2014 (ClinicalTrials.gov NCT01810614) explored the correlation between diet and markers of ADPKD. The goal of the trial was to test if changes in sodium, protein, acid precursors, and water intake can affect cyst growth. In this pre-post feasibility study, 12 adults diagnosed with ADPKD were given a diet with lower amounts of sodium and protein, and higher amounts of fruits, starchy vegetables, and water, to which the majority of the patients was able to adhere. The subjects had a trend for a decrease in systolic blood pressure potentially resulting from lower sodium intake. Furthermore, this diet reduced proton excretion in the urine. A manuscript summarizing the results of the trial mentioned that higher water intake led to lower plasma and urine osmolality, which in turn affected vasopressin by lowering its levels in plasma (Taylor et al., 2017).

Based on the recent findings, we can conclude that managing dietary intake of salt, water, protein, glucose, and other nutrients can help slow PKD progression and potentially facilitate medical treatments and improve long-term outcomes of the disease. There still are many questions related to the mechanisms of nutrients’ effects in PKD, but a solid base of evidence has been collected for preclinical and clinical trials of different dietary interventions.

## Inflammation and Microbiome in Pkd

Many studies have reported abnormal expression of genes encoding factors of the immune response, and overall activation of the immune system and infiltration of immune cells in ADPKD (de Almeida et al., 2016). One of the current hypotheses suggests that the events associated with inflammation and activation of the immune system may promote PKD; for instance, it has been shown that M2-like macrophages can promote ARPKD progression in *cpk* mice by stimulating cyst cell proliferation, cyst growth, and fibrosis (Swenson-Fields et al., 2013). Cytokines (small proteins, peptides, or glycoproteins that mediate and regulate immunity and inflammation) have also been employed in PKD development. A widely studied chemokine important for PKD, which is also involved in the development of other renal disorders, is MCP-1 (monocyte chemoattractant protein-1). МСР-1 is one of the key regulators of monocyte and macrophage migration and infiltration, and it was shown that the expression of MCP-1 is increased in urine in most patients with ADPКD, as well as in cystic fluid obtained from nephrectomy specimens (Zheng et al., 2003). Urinary MCP-1 was suggested to be a biomarker of ADPKD, and a key factor in the development of ADPKD therapy (Zheng et al., 2003). Expression of MCP-1 is controlled by TNF-α (tumor necrosis factor alpha), which is a powerful pro-inflammatory inductor that is implicated in many signaling cascades relevant for PKD (Zhou et al., 2015). TNF-α levels increase in cystic kidneys during macrophage infiltration, and it was suggested that this inflammatory cytokine may promote cyst growth and expansion during PKD progression (Zhou et al., 2015).

Innate immune cells are responsible for maintaining homeostasis and functional recovery of the kidney after injury, but in the early stages of kidney disease, activation of immune cells, and of macrophages in particular, can lead to tissue damage (Dong et al., 2007; Park et al., 2020). In PKD, changes in the innate immune response occur at early stages of the disease (Zimmerman et al., 2019b). Unfortunately, although congenital kidney immune cells are mostly evolutionarily conserved, there are significant discrepancies that make it difficult to extrapolate data from animal models to humans (Zimmerman et al., 2019a). For example, a set of immune cells shown to be activated in patients with PKD (i.e., non-specific and infiltrating macrophages, mast cells, neutrophils, B cells, and different types of T-cells) are different from cells activated in mouse models (Zimmerman et al., 2020). Thus, the discrepancies in activation of immune cells greatly complicate the study of the role of individual components of immunity in animal models and humans.

It is hypothesized that adaptive immune response (humoral response mediated by B-lymphocytes and antibodies, and cell-mediated response carried by Т-lymphocytes) may also play a role in the pathology of ADPKD. Specifically, it has been proposed that activation of adaptive immunity can be used as a biomarker for ADPKD: increased levels of interleukins (IL-23, IL-6, and, in particular, IL-17) in serum and urine in ADPKD patients and enhanced expression of SEMA7A (Semaphorin 7A) protein on immune cells (Soleimani et al., 2015; Lee et al., 2018). Interestingly, B-lymphoblastoid cells (LCL) obtained from ADPKD patients express genes encoding PC1 and PC2, and their T-cells also express PC1 and PC2, although at a lower level compared with LCL (Aguiari et al., 2004; Magistroni et al., 2019). Furthermore, in patients carrying the *PKD2* mutation, Ca^2+^ signaling was found to be impaired in circulating T-cells; T-cells derived from both patients with *PKD1* and *PKD2* mutations demonstrated increased proliferation and increased homotypic T-cell aggregation (Magistroni et al., 2019). To date, there are still many uncertainties about the participation of cells of adaptive and innate immunity in PKD. The most complete and up-to-date information on this topic, highlighting a link between PC1-PC2 complex and immune response in ADPKD, is presented in an excellent review by Zimmerman et al. (2020).

The immune system is tightly connected with the microbiome: The symbiotic relationship between the components of the immune system and the microbiota contributes to the development of an adequate response of the body to the disease-related changes (Li and Tang, 2018). The microbiome-related investigative focus is just emerging in PKD, and as with any microbiome research, it poses difficulties. For the first time, Yacoub et al. analyzed the correlation of microbiome and eGFR in patients with PKD (Yacoub et al., 2019). The patients were divided into three groups depending on their eGFR, and step-wise microbiome changes were detected in accordance with renal function. The relative prevalence of lactic acid bacteria *Lactobacillus iners* was increased in patients with eGFR <45ml/min (Yacoub et al., 2019). The human microbiome consists of 50 trillion microorganisms which work cooperatively, and any disbalance in the microbiome leads to disturbances in overall homeostasis, and vice versa. Unfortunately, in the case of PKD, it is difficult to investigate specific changes in the microbiota because of the many confounding factors that accompany PKD (hypertension, kidney failure, nephrolithiasis, and others), which can potentially alter the gut microbiota (Bergmann, 2019). Therefore, it is necessary to carefully select patients for these studies, and more large-scale research is needed in this emerging area.

## Conclusion

Despite the availability of tolvaptan for ADPKD, it is imperative to keep searching for new, side-effect free treatments that would reduce the suffering of the PKD patients. Pathophysiology of the PKD is extremely complex and includes numerous interconnected modalities and molecular mechanisms. In addition to the well-known defects in PC1 and PC2 in ADPKD and dysfunction of the FPC in ARPKD, there are multiple other pathways that are affected in these disease states. Among the emerging promising concepts in PKD, metabolic alterations, including modulation of mitochondrial bioenergetics, have been established to be important for cyst growth. Some beneficial dietary recommendations are already in place for patients with PKD, and several novel clinical trials focusing on dietary interventions (such as PREVENT-ADPKD, DRINK, and Keto-ADPKD) are ongoing or have been recently completed. It is important to develop a sustainable, patient-friendly diet, which can be followed not only during therapy, but also throughout life. We are hopeful that novel studies will provide patients with effective non-pharmacological treatments complementary to drug-based approaches. Since PKD is caused by a number of established mutations, gene therapy could be logically suggested as a means to alleviate disease progression. Unfortunately, current approaches in gene therapy are far from bedside, from both research and ethics standpoints. One of the least explored and most intriguing areas of PKD research is the immunology of this disease state, and especially the influence of the microbiome and gut-kidney interactions on cyst development. We can cautiously hope that novel discoveries in immunology and microbiology will help us solve the mystery of the complex events associated with PKD. PKD is a multisystem multifactorial disease that affects the ion homeostasis, immune system, microbiome, hormones, and a plethora of other processes; an integrated multi-angle approach is required to find new comprehensive tactics to alleviate its course without causing serious complications.

## Author Contributions

All authors participated in the writing of the manuscript, reviewed, edited, and approved the final version. VV and RS have contributed equally to this work.

## Funding

This work was supported by the NIDDK R00DK105160, NHLBI R01HL148114, and the PKD Foundation award 221G18a (all to DI).

## Conflict of Interest

The authors declare that the research was conducted in the absence of any commercial or financial relationships that could be construed as a potential conflict of interest.

## Publisher’s Note

All claims expressed in this article are solely those of the authors and do not necessarily represent those of their affiliated organizations, or those of the publisher, the editors and the reviewers. Any product that may be evaluated in this article, or claim that may be made by its manufacturer, is not guaranteed or endorsed by the publisher.

## References

[ref1] Aguiari G. Banzi M. Gessi S. Cai Y. Zeggio E. Manzati E.. (2004). Deficiency of polycystin-2 reduces Ca2^+^ channel activity and cell proliferation in ADPKD lymphoblastoid cells. FASEB J. 18, 884–886. 10.1096/fj.03-0687fje, PMID: 15001556

[ref2] Aihara M. Fujiki H. Mizuguchi H. Hattori K. Ohmoto K. Ishikawa M.. (2014). Tolvaptan delays the onset of end-stage renal disease in a polycystic kidney disease model by suppressing increases in kidney volume and renal injury. J. Pharmacol. Exp. Ther. 349, 258–267. 10.1124/jpet.114.213256, PMID: 24570071

[ref3] Alzarka B. Morizono H. Bollman J. W. Kim D. Guay-Woodford L. M. (2017). Design and implementation of the Hepatorenal Fibrocystic Disease Core Center Clinical Database: A centralized resource for characterizing autosomal recessive polycystic kidney disease and other hepatorenal fibrocystic diseases. Front. Pediatr. 5:80. 10.3389/fped.2017.00080, PMID: 28473971 PMC5397503

[ref4] Andries A. Daenen K. Jouret F. Bammens B. Mekahli D. Van Schepdael A. (2019). Oxidative stress in autosomal dominant polycystic kidney disease: player and/or early predictor for disease progression? Pediatr. Nephrol. 34, 993–1008. 10.1007/s00467-018-4004-5, PMID: 30105413

[ref5] Anyatonwu G. I. Estrada M. Tian X. Somlo S. Ehrlich B. E. (2007). Regulation of ryanodine receptor-dependent calcium signaling by polycystin-2. Proc. Natl. Acad. Sci. U. S. A. 104:17404231, 6454–6459. 10.1073/pnas.0610324104 PMC1851053 17404231

[ref6] Banerjee T. Crews D. C. Wesson D. E. Tilea A. M. Saran R. Rios-Burrows N.. (2015). High dietary acid load predicts ESRD among adults with CKD. J. Am. Soc. Nephrol. 26, 1693–1700. 10.1681/ASN.2014040332, PMID: 25677388 PMC4483581

[ref7] Beck Gooz M. Maldonado E. N. Dang Y. Amria M. Y. Higashiyama S. Abboud H. E.. (2014). ADAM17 promotes proliferation of collecting duct kidney epithelial cells through ERK activation and increased glycolysis in polycystic kidney disease. Am. J. Physiol. Renal Physiol. 307, F551–F559. 10.1152/ajprenal.00218.2014, PMID: 24899059 PMC4154111

[ref8] Bergmann C. (2019). Early and severe polycystic kidney disease and related ciliopathies: An emerging field of interest. Nephron 141, 50–60. 10.1159/000493532, PMID: 30359986

[ref9] Boehlke C. Kotsis F. Patel V. Braeg S. Voelker H. Bredt S.. (2010). Primary cilia regulate mTORC1 activity and cell size through Lkb1. Nat. Cell Biol. 12, 1115–1122. 10.1038/ncb2117, PMID: 20972424 PMC3390256

[ref10] Brosnahan G. M. Abebe K. Z. Moore C. G. Bae K. T. Braun W. E. Chapman A. B.. (2018). Determinants of progression in early autosomal dominant polycystic kidney disease: is it blood pressure or renin-angiotensin-aldosterone-system blockade? Curr. Hypertens. Rev. 14, 39–47. 10.2174/1573402114666180322110209, PMID: 29564978 PMC6063360

[ref12] Burgmaier K. Brinker L. Erger F. Beck B. B. Benz M. R. Bergmann C.. (2021). Refining genotype-phenotype correlations in 304 patients with autosomal recessive polycystic kidney disease and PKHD1 gene variants. Kidney Int. 100, 650–47. 10.1016/j.kint.2021.04.019, PMID: 33940108

[ref13] Cabrita I. Talbi K. Kunzelmann K. Schreiber R. (2021). Loss of PKD1 and PKD2 share common effects on intracellular Ca(2+) signaling. Cell Calcium 97:102413. 10.1016/j.ceca.2021.102413, PMID: 33915319

[ref14] Cai Y. Fedeles S. V. Dong K. Anyatonwu G. Onoe T. Mitobe M.. (2014). Altered trafficking and stability of polycystins underlie polycystic kidney disease. J. Clin. Invest. 124, 5129–5144. 10.1172/JCI67273, PMID: 25365220 PMC4348948

[ref15] Cantero Mdel R. Velazquez I. F. Streets A. J. Ong A. C. Cantiello H. F. (2015). The cAMP signaling pathway and direct protein kinase a phosphorylation regulate polycystin-2 (TRPP2) channel function. J. Biol. Chem. 290, 23888–23896. 10.1074/jbc.M115.661082, PMID: 26269590 PMC4583051

[ref16] Casteleijn N. F. Blais J. D. Chapman A. B. Czerwiec F. S. Devuyst O. Higashihara E.. (2017). Tolvaptan and kidney pain in patients with autosomal dominant polycystic kidney disease: secondary analysis from a randomized controlled trial. Am. J. Kidney Dis. 69, 210–219. 10.1053/j.ajkd.2016.08.028, PMID: 27856088 PMC5497700

[ref17] Cebotaru V. Cebotaru L. Kim H. Chiaravalli M. Boletta A. Qian F.. (2014). Polycystin-1 negatively regulates polycystin-2 expression via the aggresome/autophagosome pathway. J. Biol. Chem. 289, 6404–6414. 10.1074/jbc.M113.501205, PMID: 24459142 PMC3945307

[ref18] Chacon-Heszele M. F. Choi S. Y. Zuo X. Baek J. I. Ward C. Lipschutz J. H. (2014). The exocyst and regulatory GTPases in urinary exosomes. Physiol. Rep. 2:e12116. 10.14814/phy2.12116, PMID: 25138791 PMC4246586

[ref19] Chang M. Y. Hsieh C. Y. Lin C. Y. Chen T. D. Yang H. Y. Chen K. H.. (2018). Effect of celastrol on the progression of polycystic kidney disease in a Pkd1-deficient mouse model. Life Sci. 212, 70–79. 10.1016/j.lfs.2018.09.047, PMID: 30268856

[ref20] Chang M. Y. Parker E. El Nahas M. Haylor J. L. Ong A. C. (2007). Endothelin B receptor blockade accelerates disease progression in a murine model of autosomal dominant polycystic kidney disease. J. Am. Soc. Nephrol. 18, 560–569. 10.1681/ASN.2006090994, PMID: 17202412

[ref21] Chen L. Zhou X. Fan L. X. Yao Y. Swenson-Fields K. I. Gadjeva M.. (2015). Macrophage migration inhibitory factor promotes cyst growth in polycystic kidney disease. J. Clin. Invest. 125, 2399–2412. 10.1172/JCI80467, PMID: 25961459 PMC4497763

[ref22] Chiaravalli M. Rowe I. Mannella V. Quilici G. Canu T. Bianchi V.. (2016). 2-Deoxy-d-glucose ameliorates PKD progression. J. Am. Soc. Nephrol. 27, 1958–1969. 10.1681/ASN.2015030231, PMID: 26534924 PMC4926967

[ref24] Cowley B. D. Jr. Grantham J. J. Muessel M. J. Kraybill A. L. Gattone V. H.(1996). Modification of disease progression in rats with inherited polycystic kidney disease. Am. J. Kidney Dis. 27, 865–879. 10.1016/S0272-6386(96)90525-9, PMID: 8651252

[ref25] De Almeida R. M. Clendenon S. G. Richards W. G. Boedigheimer M. Damore M. Rossetti S.. (2016). Transcriptome analysis reveals manifold mechanisms of cyst development in ADPKD. Hum. Genomics 10:37. 10.1186/s40246-016-0095-x, PMID: 27871310 PMC5117508

[ref27] Dell K. M. Nemo R. Sweeney W. E. Jr. Levin J. I. Frost P. Avner E. D. (2001). A novel inhibitor of tumor necrosis factor-alpha converting enzyme ameliorates polycystic kidney disease. Kidney Int. 60, 1240–1248. 10.1046/j.1523-1755.2001.00963.x, PMID: 11576338

[ref28] Delling M. Indzhykulian A. A. Liu X. Li Y. Xie T. Corey D. P.. (2016). Primary cilia are not calcium-responsive mechanosensors. Nature 531, 656–660. 10.1038/nature17426, PMID: 27007841 PMC4851444

[ref29] Doerr N. Wang Y. Kipp K. R. Liu G. Benza J. J. Pletnev V.. (2016). Regulation of polycystin-1 function by calmodulin binding. PLoS One 11:e0161525. 10.1371/journal.pone.0161525, PMID: 27560828 PMC4999191

[ref30] Dong K. Miao H. Jia X. Wu J. Wu H. Sun J.. (2019). Identification of a pathogenic mutation in a Chinese pedigree with polycystic kidney disease. Mol. Med. Rep. 19, 2671–2679. 10.3892/mmr.2019.9921, PMID: 30720121 PMC6423614

[ref31] Dong X. Swaminathan S. Bachman L. A. Croatt A. J. Nath K. A. Griffin M. D. (2007). Resident dendritic cells are the predominant TNF-secreting cell in early renal ischemia-reperfusion injury. Kidney Int. 71, 619–628. 10.1038/sj.ki.5002132, PMID: 17311071

[ref32] Douguet D. Patel A. Honore E. (2019). Structure and function of polycystins: insights into polycystic kidney disease. Nat. Rev. Nephrol. 15, 412–422. 10.1038/s41581-019-0143-6, PMID: 30948841

[ref33] Elberg D. Jayaraman S. Turman M. A. Elberg G. (2012). Transforming growth factor-beta inhibits cystogenesis in human autosomal dominant polycystic kidney epithelial cells. Exp. Cell Res. 318, 1508–1516. 10.1016/j.yexcr.2012.03.021, PMID: 22504005

[ref34] El-Damanawi R. Lee M. Harris T. Cowley L. B. Bond S. Pavey H.. (2020). High water vs. ad libitum water intake for autosomal dominant polycystic kidney disease: a randomized controlled feasibility trial. QJM 113, 258–265. 10.1093/qjmed/hcz278, PMID: 31665476 PMC7133783

[ref35] El-Damanawi R. Lee M. Harris T. Mader L. B. Bond S. Pavey H.. (2018). Randomised controlled trial of high versus ad libitum water intake in patients with autosomal dominant polycystic kidney disease: rationale and design of the DRINK feasibility trial. BMJ Open 8:e022859. 10.1136/bmjopen-2018-022859, PMID: 29743334 PMC5942404

[ref36] Erickson K. F. Chertow G. M. Goldhaber-Fiebert J. D. (2013). Cost-effectiveness of tolvaptan in autosomal dominant polycystic kidney disease. Ann. Intern. Med. 159, 382–389. 10.7326/0003-4819-159-6-201309170-00004, PMID: 24042366 PMC3981098

[ref37] Fitzgibbon W. R. Dang Y. Bunni M. A. Baicu C. F. Zile M. R. Mullick A. E.. (2018). Attenuation of accelerated renal cystogenesis in Pkd1 mice by renin-angiotensin system blockade. Am. J. Physiol. Renal Physiol. 314, F210–F218. 10.1152/ajprenal.00389.2017, PMID: 29021226 PMC5866454

[ref38] Fogelgren B. Lin S. Y. Zuo X. Jaffe K. M. Park K. M. Reichert R. J.. (2011). The exocyst protein Sec10 interacts with Polycystin-2 and knockdown causes PKD-phenotypes. PLoS Genet. 7:e1001361. 10.1371/journal.pgen.1001361, PMID: 21490950 PMC3072367

[ref39] Fu Z. J. Wang Z. Y. Xu L. Chen X. H. Li X. X. Liao W. T.. (2020). HIF-1alpha-BNIP3-mediated mitophagy in tubular cells protects against renal ischemia/reperfusion injury. Redox Biol. 36:101671. 10.1016/j.redox.2020.101671, PMID: 32829253 PMC7452120

[ref40] Gainullin V. G. Hopp K. Ward C. J. Hommerding C. J. Harris P. C. (2015). Polycystin-1 maturation requires polycystin-2 in a dose-dependent manner. J. Clin. Invest. 125, 607–620. 10.1172/JCI76972, PMID: 25574838 PMC4350419

[ref41] Gattone V. H. 2nd Kuenstler K. A. Lindemann G. W. Lu X. Cowley B. D. Jr. Rankin C. A.. (1996). Renal expression of a transforming growth factor-alpha transgene accelerates the progression of inherited, slowly progressive polycystic kidney disease in the mouse. J. Lab. Clin. Med. 127, 214–222. 10.1016/S0022-2143(96)90081-5, PMID: 8636651

[ref42] Goraya N. Simoni J. Jo C. Wesson D. E. (2012). Dietary acid reduction with fruits and vegetables or bicarbonate attenuates kidney injury in patients with a moderately reduced glomerular filtration rate due to hypertensive nephropathy. Kidney Int. 81, 86–93. 10.1038/ki.2011.313, PMID: 21881553

[ref43] Goto M. Hoxha N. Osman R. Dell K. M. (2010). The renin-angiotensin system and hypertension in autosomal recessive polycystic kidney disease. Pediatr. Nephrol. 25, 2449–2457. 10.1007/s00467-010-1621-z, PMID: 20798958

[ref44] Grieben M. Pike A. C. Shintre C. A. Venturi E. El-Ajouz S. Tessitore A.. (2017). Structure of the polycystic kidney disease TRP channel Polycystin-2 (PC2). Nat. Struct. Mol. Biol. 24, 114–122. 10.1038/nsmb.3343, PMID: 27991905

[ref45] Ha K. Nobuhara M. Wang Q. Walker R. V. Qian F. Schartner C.. (2020). The heteromeric PC-1/PC-2 polycystin complex is activated by the PC-1 N-terminus. elife 9:e60684, 33164752. 10.7554/eLife.60684 33164752 PMC7728438

[ref46] Hajarnis S. Lakhia R. Yheskel M. Williams D. Sorourian M. Liu X.. (2017). microRNA-17 family promotes polycystic kidney disease progression through modulation of mitochondrial metabolism. Nat. Commun. 8:14395. 10.1038/ncomms14395, PMID: 28205547 PMC5316862

[ref47] Hama T. Park F. (2016). Heterotrimeric G protein signaling in polycystic kidney disease. Physiol. Genomics 48, 429–445. 10.1152/physiolgenomics.00027.2016, PMID: 27199453 PMC4967223

[ref48] Hanudel M. R. Salusky I. B. Pereira R. C. Wang W. You Z. Nowak K. L.. (2019). Erythropoietin and fibroblast growth factor 23 in autosomal dominant polycystic kidney disease patients. Kidney Int. Rep. 4, 1742–1748. 10.1016/j.ekir.2019.08.010, PMID: 31844811 PMC6895647

[ref49] Hardy E. Tsiokas L. (2020). Polycystins as components of large multiprotein complexes of polycystin interactors. Cell. Signal. 72:109640. 10.1016/j.cellsig.2020.109640, PMID: 32305669 PMC7269800

[ref50] Heerspink H. L. Ritz E. (2012). Sodium chloride intake: is lower always better? J. Am. Soc. Nephrol. 23, 1136–1139. 10.1681/ASN.2012010099, PMID: 22677555

[ref51] Hian C. K. Lee C. L. Thomas W. (2016). Renin-angiotensin-aldosterone system antagonism and polycystic kidney disease progression. Nephron 134, 59–63. 10.1159/000448296, PMID: 27476173

[ref52] Hopp K. Wang X. Ye H. Irazabal M. V. Harris P. C. Torres V. E. (2015). Effects of hydration in rats and mice with polycystic kidney disease. Am. J. Physiol. Renal Physiol. 308, F261–F266. 10.1152/ajprenal.00345.2014, PMID: 25503729 PMC4312959

[ref53] Hopp K. Ward C. J. Hommerding C. J. Nasr S. H. Tuan H. F. Gainullin V. G.. (2012). Functional polycystin-1 dosage governs autosomal dominant polycystic kidney disease severity. J. Clin. Invest. 122, 4257–4273. 10.1172/JCI64313, PMID: 23064367 PMC3484456

[ref54] Hughes J. Ward C. J. Peral B. Aspinwall R. Clark K. San Millan J. L.. (1995). The polycystic kidney disease 1 (PKD1) gene encodes a novel protein with multiple cell recognition domains. Nat. Genet. 10, 151–160. 10.1038/ng0695-151, PMID: 7663510

[ref55] Ilatovskaya D. V. Levchenko V. Pavlov T. S. Isaeva E. Klemens C. A. Johnson J.. (2019). Salt-deficient diet exacerbates cystogenesis in ARPKD via epithelial sodium channel (ENaC). EBioMedicine 40, 663–674. 10.1016/j.ebiom.2019.01.006, PMID: 30745171 PMC6413684

[ref56] Ishimoto Y. Inagi R. Yoshihara D. Kugita M. Nagao S. Shimizu A.. (2017). Mitochondrial abnormality facilitates cyst formation in autosomal dominant polycystic kidney disease. Mol. Cell. Biol. 37:e00337-17. 10.1128/MCB.00337-17, PMID: 28993480 PMC5705822

[ref57] Jager S. Handschin C. St-Pierre J. Spiegelman B. M. (2007). AMP-activated protein kinase (AMPK) action in skeletal muscle via direct phosphorylation of PGC-1alpha. Proc. Natl. Acad. Sci. U. S. A. 104:17609368, 12017–12022. 10.1073/pnas.0705070104. PMC1924552 17609368

[ref58] Kanai T. Shiizaki K. Betsui H. Aoyagi J. Yamagata T. (2018). A decreased soluble Klotho level with normal eGFR, FGF23, serum phosphate, and FEP in an ADPKD patient with enlarged kidneys due to multiple cysts. CEN Case Rep. 7, 259–263. 10.1007/s13730-018-0339-9, PMID: 29767399 PMC6181895

[ref59] Kanhai A. A. Bange H. Verburg L. Dijkstra K. L. Price L. S. Peters D. J. M.. (2020). Renal cyst growth is attenuated by a combination treatment of tolvaptan and pioglitazone, while pioglitazone treatment alone is not effective. Sci. Rep. 10:1672. 10.1038/s41598-020-58382-z, PMID: 32015419 PMC6997373

[ref60] Kim I. Ding T. Fu Y. Li C. Cui L. Li A.. (2009). Conditional mutation of Pkd2 causes cystogenesis and upregulates beta-catenin. J. Am. Soc. Nephrol. 20, 2556–2569. 10.1681/ASN.2009030271, PMID: 19939939 PMC2794231

[ref61] Kim H. Park S. Jhee J. H. Yun H. R. Park J. T. Han S. H.. (2019). Urinary angiotensinogen level is associated with potassium homeostasis and clinical outcome in patients with polycystic kidney disease: a prospective cohort study. BMC Nephrol. 20:104. 10.1186/s12882-019-1292-3, PMID: 30909873 PMC6434770

[ref62] Kipp K. R. Rezaei M. Lin L. Dewey E. C. Weimbs T. (2016). A mild reduction of food intake slows disease progression in an orthologous mouse model of polycystic kidney disease. Am. J. Physiol. Renal Physiol. 310, F726–F731. 10.1152/ajprenal.00551.2015, PMID: 26764208 PMC4835927

[ref63] Klahr S. Breyer J. A. Beck G. J. Dennis V. W. Hartman J. A. Roth D.. (1995). Dietary protein restriction, blood pressure control, and the progression of polycystic kidney disease. Modification of Diet in Renal Disease Study Group. J. Am. Soc. Nephrol. 5, 2037–2047. 10.1681/ASN.V5122037 7579052

[ref64] Kleene S. J. Kleene N. K. (2017). The native TRPP2-dependent channel of murine renal primary cilia. Am. J. Physiol. Renal Physiol. 312, F96–F108. 10.1152/ajprenal.00272.2016, PMID: 27760766 PMC5283891

[ref65] Kocer D. Karakukcu C. Ozturk F. Eroglu E. Kocyigit I. (2016). Evaluation of fibrosis markers: Apelin and transforming growth factor-beta1 in autosomal dominant polycystic kidney disease patients. Ther. Apher. Dial. 20, 517–522. 10.1111/1744-9987.12412, PMID: 26991810

[ref66] Kocyigit I. Eroglu E. Kaynar A. S. Kocer D. Kargi S. Zararsiz G.. (2019). The association of endothelin-1 levels with renal survival in polycystic kidney disease patients. J. Nephrol. 32, 83–91. 10.1007/s40620-018-0514-2, PMID: 30022320

[ref67] Konvalinka A. Batruch I. Tokar T. Dimitromanolakis A. Reid S. Song X.. (2016). Quantification of angiotensin II-regulated proteins in urine of patients with polycystic and other chronic kidney diseases by selected reaction monitoring. Clin. Proteomics 13:16. 10.1186/s12014-016-9117-x, PMID: 27499720 PMC4974759

[ref68] Kuo I. Y. Brill A. L. Lemos F. O. Jiang J. Y. Falcone J. L. Kimmerling E. P.. (2019). Polycystin 2 regulates mitochondrial ca(2+) signaling, bioenergetics, and dynamics through mitofusin 2. Sci. Signal. 12:eaat7397. 10.1126/scisignal.aat7397, PMID: 31064883 PMC6855602

[ref69] Kuo I. Y. Chapman A. B. (2020). Polycystins, ADPKD, and cardiovascular disease. Kidney Int. Rep. 5, 396–406. 10.1016/j.ekir.2019.12.007, PMID: 32274448 PMC7136326

[ref70] Kurbegovic A. Kim H. Xu H. Yu S. Cruanes J. Maser R. L.. (2014). Novel functional complexity of polycystin-1 by GPS cleavage in vivo: role in polycystic kidney disease. Mol. Cell. Biol. 34, 3341–3353. 10.1128/MCB.00687-14, PMID: 24958103 PMC4135549

[ref71] Lakhia R. Yheskel M. Flaten A. Quittner-Strom E. B. Holland W. L. Patel V. (2018). PPARalpha agonist fenofibrate enhances fatty acid beta-oxidation and attenuates polycystic kidney and liver disease in mice. Am. J. Physiol. Renal Physiol. 314, F122–F131. 10.1152/ajprenal.00352.2017, PMID: 28903946 PMC5866355

[ref72] Lakshmipathi J. Gao Y. Hu C. Stuart D. Genzen J. Ramkumar N.. (2020). Nephron-specific disruption of polycystin-1 induces cyclooxygenase-2-mediated blood pressure reduction independent of cystogenesis. J. Am. Soc. Nephrol. 31, 1243–1254. 10.1681/ASN.2019090934, PMID: 32300065 PMC7269346

[ref73] Lee Y. Blount K. L. Dai F. Thompson S. Scher J. K. Bitterman S.. (2018). Semaphorin 7A in circulating regulatory T cells is increased in autosomal-dominant polycystic kidney disease and decreases with tolvaptan treatment. Clin. Exp. Nephrol. 22, 906–916. 10.1007/s10157-018-1542-x, PMID: 29453607

[ref74] Lee K. L. Guevarra M. D. Nguyen A. M. Chua M. C. Wang Y. Jacobs C. R. (2015). The primary cilium functions as a mechanical and calcium signaling nexus. Cilia 4:7. 10.1186/s13630-015-0016-y, PMID: 26029358 PMC4448211

[ref75] Leonhard W. N. Kunnen S. J. Plugge A. J. Pasternack A. Jianu S. B. Veraar K.. (2016). Inhibition of activin signaling slows progression of polycystic kidney disease. J. Am. Soc. Nephrol. 27, 3589–3599. 10.1681/ASN.2015030287, PMID: 27020852 PMC5118473

[ref76] Li D. Y. Tang W. H. W. (2018). Contributory role of gut microbiota and their metabolites toward cardiovascular complications in chronic kidney disease. Semin. Nephrol. 38, 193–205. 10.1016/j.semnephrol.2018.01.008, PMID: 29602401 PMC5881581

[ref77] Lian X. Wu X. Li Z. Zhang Y. Song K. Cai G.. (2019). The combination of metformin and 2-deoxyglucose significantly inhibits cyst formation in miniature pigs with polycystic kidney disease. Br. J. Pharmacol. 176, 711–724. 10.1111/bph.14558, PMID: 30515768 PMC6365356

[ref78] Lin F. Hiesberger T. Cordes K. Sinclair A. M. Goldstein L. S. Somlo S.. (2003). Kidney-specific inactivation of the KIF3A subunit of kinesin-II inhibits renal ciliogenesis and produces polycystic kidney disease. Proc. Natl. Acad. Sci. U. S. A. 100, 5286–5291. 10.1073/pnas.0836980100, PMID: 12672950 PMC154337

[ref79] Liu W. Xu S. Woda C. Kim P. Weinbaum S. Satlin L. M. (2003). Effect of flow and stretch on the [Ca2^+^]i response of principal and intercalated cells in cortical collecting duct. Am. J. Physiol. Renal Physiol. 285, F998–F1012. 10.1152/ajprenal.00067.2003, PMID: 12837680

[ref80] Loghman-Adham M. Soto C. E. Inagami T. Cassis L. (2004). The intrarenal renin-angiotensin system in autosomal dominant polycystic kidney disease. Am. J. Physiol. Renal Physiol. 287, F775–F788. 10.1152/ajprenal.00370.2003, PMID: 15187005

[ref81] Loghman-Adham M. Soto C. E. Inagami T. Sotelo-Avila C. (2005). Expression of components of the renin-angiotensin system in autosomal recessive polycystic kidney disease. J. Histochem. Cytochem. 53, 979–988. 10.1369/jhc.4A6494.2005, PMID: 15879580

[ref82] Ma M. Gallagher A. R. Somlo S. (2017). Ciliary mechanisms of cyst formation in polycystic kidney disease. Cold Spring Harb. Perspect. Biol. 9:a028209. 10.1101/cshperspect.a028209, PMID: 28320755 PMC5666631

[ref83] Ma M. Tian X. Igarashi P. Pazour G. J. Somlo S. (2013). Loss of cilia suppresses cyst growth in genetic models of autosomal dominant polycystic kidney disease. Nat. Genet. 45, 1004–1012. 10.1038/ng.2715, PMID: 23892607 PMC3758452

[ref84] Magistroni R. Mangolini A. Guzzo S. Testa F. Rapana M. R. Mignani R.. (2019). TRPP2 dysfunction decreases ATP-evoked calcium, induces cell aggregation and stimulates proliferation in T lymphocytes. BMC Nephrol. 20:355. 10.1186/s12882-019-1540-6, PMID: 31514750 PMC6743124

[ref85] Mahajan A. Simoni J. Sheather S. J. Broglio K. R. Rajab M. H. Wesson D. E. (2010). Daily oral sodium bicarbonate preserves glomerular filtration rate by slowing its decline in early hypertensive nephropathy. Kidney Int. 78, 303–309. 10.1038/ki.2010.129, PMID: 20445497

[ref86] Malekshahabi T. Khoshdel Rad N. Serra A. L. Moghadasali R. (2019). Autosomal dominant polycystic kidney disease: disrupted pathways and potential therapeutic interventions. J. Cell. Physiol. 234, 12451–12470. 10.1002/jcp.28094, PMID: 30644092 PMC13484091

[ref87] Mei K. Li Y. Wang S. Shao G. Wang J. Ding Y.. (2018). Cryo-EM structure of the exocyst complex. Nat. Struct. Mol. Biol. 25, 139–146. 10.1038/s41594-017-0016-2, PMID: 29335562 PMC5971111

[ref88] Melchionda S. Palladino T. Castellana S. Giordano M. Benetti E. De Bonis P.. (2016). Expanding the mutation spectrum in 130 probands with ARPKD: identification of 62 novel PKHD1 mutations by sanger sequencing and MLPA analysis. J. Hum. Genet. 61, 811–821. 10.1038/jhg.2016.58, PMID: 27225849

[ref89] Menezes L. F. Germino G. G. (2019). The pathobiology of polycystic kidney disease from a metabolic viewpoint. Nat. Rev. Nephrol. 15, 735–749. 10.1038/s41581-019-0183-y, PMID: 31488901

[ref90] Menezes L. F. Lin C. C. Zhou F. Germino G. G. (2016). Fatty acid oxidation is impaired in an orthologous mouse model of autosomal dominant polycystic kidney disease. EBioMedicine 5, 183–192. 10.1016/j.ebiom.2016.01.027, PMID: 27077126 PMC4816756

[ref91] Middleton J. P. Lehrich R. W. (2014). Prescriptions for dietary sodium in patients with chronic kidney disease: how will this shake out? Kidney Int. 86, 457–459. 10.1038/ki.2014.124, PMID: 25168493

[ref92] Nagao S. Nishii K. Katsuyama M. Kurahashi H. Marunouchi T. Takahashi H.. (2006). Increased water intake decreases progression of polycystic kidney disease in the PCK rat. J. Am. Soc. Nephrol. 17, 2220–2227. 10.1681/ASN.2006030251, PMID: 16807403

[ref93] Natoli T. A. Smith L. A. Rogers K. A. Wang B. Komarnitsky S. Budman Y.. (2010). Inhibition of glucosylceramide accumulation results in effective blockade of polycystic kidney disease in mouse models. Nat. Med. 16, 788–792. 10.1038/nm.2171, PMID: 20562878 PMC3660226

[ref94] Nauli S. M. Alenghat F. J. Luo Y. Williams E. Vassilev P. Li X.. (2003). Polycystins 1 and 2 mediate mechanosensation in the primary cilium of kidney cells. Nat. Genet. 33, 129–137. 10.1038/ng1076, PMID: 12514735

[ref95] Nigro E. A. Distefano G. Chiaravalli M. Matafora V. Castelli M. Pesenti Gritti A.. (2019). Polycystin-1 regulates actomyosin contraction and the cellular response to extracellular stiffness. Sci. Rep. 9:16640. 10.1038/s41598-019-53061-0, PMID: 31719603 PMC6851149

[ref96] Nikonova A. S. Deneka A. Y. Kiseleva A. A. Korobeynikov V. Gaponova A. Serebriiskii I. G.. (2018). Ganetespib limits ciliation and cystogenesis in autosomal-dominant polycystic kidney disease (ADPKD). FASEB J. 32, 2735–2746. 10.1096/fj.201700909R, PMID: 29401581 PMC5901382

[ref97] Nims N. Vassmer D. Maser R. L. (2003). Transmembrane domain analysis of polycystin-1, the product of the polycystic kidney disease-1 (PKD1) gene: evidence for 11 membrane-spanning domains. Biochemistry 42, 13035–13048. 10.1021/bi035074c, PMID: 14596619

[ref98] Noh M. R. Jang H. S. Song D. K. Lee S. R. Lipschutz J. H. Park K. M.. (2018). Downregulation of exocyst Sec10 accelerates kidney tubule cell recovery through enhanced cell migration. Biochem. Biophys. Res. Commun. 496, 309–315. 10.1016/j.bbrc.2018.01.013, PMID: 29326040

[ref99] Nowak K. L. Gitomer B. Farmer-Bailey H. Wang W. Malaczewski M. Klawitter J.. (2019). Mineralocorticoid antagonism and vascular function in early autosomal dominant polycystic kidney disease: a randomized controlled trial. Am. J. Kidney Dis. 74, 213–223. 10.1053/j.ajkd.2018.12.037, PMID: 30803706 PMC6660387

[ref100] Nowak K. L. Wang W. Farmer-Bailey H. Gitomer B. Malaczewski M. Klawitter J.. (2018). Vascular dysfunction, oxidative stress, and inflammation in autosomal dominant polycystic kidney disease. Clin. J. Am. Soc. Nephrol. 13, 1493–1501. 10.2215/CJN.05850518, PMID: 30228110 PMC6218833

[ref101] O’donnell M. J. Yusuf S. Mente A. Gao P. Mann J. F. Teo K.. (2011). Urinary sodium and potassium excretion and risk of cardiovascular events. JAMA 306, 2229–2238. 10.1001/jama.2011.1729, PMID: 22110105

[ref102] Ogborn M. R. Sareen S. (1995). Amelioration of polycystic kidney disease by modification of dietary protein intake in the rat. J. Am. Soc. Nephrol. 6, 1649–1654. 10.1681/ASN.V661649, PMID: 8749693

[ref103] Omede F. Zhang S. Johnson C. Daniel E. Zhang Y. Fields T. A.. (2020). Dietary phosphate restriction attenuates polycystic kidney disease in mice. Am. J. Physiol. Renal Physiol. 318, F35–F42. 10.1152/ajprenal.00282.2019, PMID: 31682174 PMC6985825

[ref104] Outeda P. Menezes L. Hartung E. A. Bridges S. Zhou F. Zhu X.. (2017). A novel model of autosomal recessive polycystic kidney questions the role of the fibrocystin C-terminus in disease mechanism. Kidney Int. 92, 1130–1144. 10.1016/j.kint.2017.04.027, PMID: 28729032 PMC6005173

[ref105] Padovano V. Kuo I. Y. Stavola L. K. Aerni H. R. Flaherty B. J. Chapin H. C.. (2017). The polycystins are modulated by cellular oxygen-sensing pathways and regulate mitochondrial function. Mol. Biol. Cell 28, 261–269. 10.1091/mbc.E16-08-0597, PMID: 27881662 PMC5231895

[ref106] Pala R. Alomari N. Nauli S. M. (2017). Primary cilium-dependent signaling mechanisms. Int. J. Mol. Sci. 18:2272. 10.3390/ijms18112272, PMID: 29143784 PMC5713242

[ref107] Park K. M. (2018). Can tissue cilia lengths and urine cilia proteins be markers of kidney diseases? Chonnam Med. J. 54, 83–89. 10.4068/cmj.2018.54.2.83, PMID: 29854673 PMC5972129

[ref108] Park J. G. Na M. Kim M. G. Park S. H. Lee H. J. Kim D. K.. (2020). Immune cell composition in normal human kidneys. Sci. Rep. 10:15678. 10.1038/s41598-020-80120-8, PMID: 32973321 PMC7515917

[ref109] Parnell S. C. Magenheimer B. S. Maser R. L. Rankin C. A. Smine A. Okamoto T.. (1998). The polycystic kidney disease-1 protein, polycystin-1, binds and activates heterotrimeric G-proteins in vitro. Biochem. Biophys. Res. Commun. 251, 625–631. 10.1006/bbrc.1998.9514, PMID: 9792824

[ref110] Patch C. Charlton J. Roderick P. J. Gulliford M. C. (2011). Use of antihypertensive medications and mortality of patients with autosomal dominant polycystic kidney disease: a population-based study. Am. J. Kidney Dis. 57, 856–862. 10.1053/j.ajkd.2011.01.023, PMID: 21458899

[ref111] Phillips J. K. Hopwood D. Loxley R. A. Ghatora K. Coombes J. D. Tan Y. S.. (2007). Temporal relationship between renal cyst development, hypertension and cardiac hypertrophy in a new rat model of autosomal recessive polycystic kidney disease. Kidney Blood Press. Res. 30, 129–144. 10.1159/000101828, PMID: 17446713

[ref112] Picco A. Irastorza-Azcarate I. Specht T. Boke D. Pazos I. Rivier-Cordey A. S.. (2017). The in vivo architecture of the exocyst provides structural basis for exocytosis. Cell 168, 400.e418–412.e418. 10.1016/j.cell.2017.01.004, PMID: 28129539

[ref113] Podrini C. Cassina L. Boletta A. (2020). Metabolic reprogramming and the role of mitochondria in polycystic kidney disease. Cell. Signal. 67:109495. 10.1016/j.cellsig.2019.109495, PMID: 31816397

[ref114] Podrini C. Rowe I. Pagliarini R. Costa A. S. H. Chiaravalli M. Di Meo I.. (2018). Dissection of metabolic reprogramming in polycystic kidney disease reveals coordinated rewiring of bioenergetic pathways. Commun. Biol. 1:194. 10.1038/s42003-018-0200-x, PMID: 30480096 PMC6240072

[ref115] Puder S. Fischer T. Mierke C. T. (2019). The transmembrane protein fibrocystin/polyductin regulates cell mechanics and cell motility. Phys. Biol. 16:066006. 10.1088/1478-3975/ab39fa, PMID: 31398719

[ref116] Qian F. Germino F. J. Cai Y. Zhang X. Somlo S. Germino G. G. (1997). PKD1 interacts with PKD2 through a probable coiled-coil domain. Nat. Genet. 16, 179–183. 10.1038/ng0697-179, PMID: 9171830

[ref117] Raina R. Lou L. Berger B. Vogt B. Do A. S. Cunningham R.. (2016). Relationship of urinary endothelin-1 with estimated glomerular filtration rate in autosomal dominant polycystic kidney disease: a pilot cross-sectional analysis. BMC Nephrol. 17:22. 10.1186/s12882-016-0232-8, PMID: 26923419 PMC4770683

[ref118] Rees S. Kittikulsuth W. Roos K. Strait K. A. Van Hoek A. Kohan D. E. (2014). Adenylyl cyclase 6 deficiency ameliorates polycystic kidney disease. J. Am. Soc. Nephrol. 25, 232–237. 10.1681/ASN.2013010077, PMID: 24158982 PMC3904559

[ref119] Reif G. A. Yamaguchi T. Nivens E. Fujiki H. Pinto C. S. Wallace D. P. (2011). Tolvaptan inhibits ERK-dependent cell proliferation, Cl^−^ secretion, and in vitro cyst growth of human ADPKD cells stimulated by vasopressin. Am. J. Physiol. Renal Physiol. 301, F1005–F1013. 10.1152/ajprenal.00243.2011, PMID: 21816754 PMC3213906

[ref120] Riwanto M. Kapoor S. Rodriguez D. Edenhofer I. Segerer S. Wuthrich R. P. (2016). Inhibition of aerobic glycolysis attenuates disease progression in polycystic kidney disease. PLoS One 11:e0146654. 10.1371/journal.pone.0146654, PMID: 26752072 PMC4708993

[ref121] Rogers K. A. Moreno S. E. Smith L. A. Husson H. Bukanov N. O. Ledbetter S. R.. (2016). Differences in the timing and magnitude of Pkd1 gene deletion determine the severity of polycystic kidney disease in an orthologous mouse model of ADPKD. Physiol. Rep. 4:e12846. 10.14814/phy2.12846, PMID: 27356569 PMC4926022

[ref122] Rossi A. Pizzo P. Filadi R. (2019). Calcium, mitochondria and cell metabolism: A functional triangle in bioenergetics. Biochim. Biophys. Acta, Mol. Cell Res. 1866, 1068–1078. 10.1016/j.bbamcr.2018.10.016 30982525

[ref123] Rowe I. Chiaravalli M. Mannella V. Ulisse V. Quilici G. Pema M.. (2013). Defective glucose metabolism in polycystic kidney disease identifies a new therapeutic strategy. Nat. Med. 19, 488–493. 10.1038/nm.3092, PMID: 23524344 PMC4944011

[ref124] Ruh H. Salonikios T. Fuchser J. Schwartz M. Sticht C. Hochheim C.. (2013). MALDI imaging MS reveals candidate lipid markers of polycystic kidney disease. J. Lipid Res. 54, 2785–2794. 10.1194/jlr.M040014, PMID: 23852700 PMC3770091

[ref125] Rydholm S. Zwartz G. Kowalewski J. M. Kamali-Zare P. Frisk T. Brismar H. (2010). Mechanical properties of primary cilia regulate the response to fluid flow. Am. J. Physiol. Renal Physiol. 298, F1096–F1102. 10.1152/ajprenal.00657.2009, PMID: 20089672

[ref126] Saburi S. Hester I. Fischer E. Pontoglio M. Eremina V. Gessler M.. (2008). Loss of Fat4 disrupts PCP signaling and oriented cell division and leads to cystic kidney disease. Nat. Genet. 40, 1010–1015. 10.1038/ng.179, PMID: 18604206

[ref127] Sagar P. S. Zhang J. Luciuk M. Mannix C. Wong A. T. Y. Rangan G. K. (2019). Increased water intake reduces long-term renal and cardiovascular disease progression in experimental polycystic kidney disease. PLoS One 14:e0209186. 10.1371/journal.pone.0209186, PMID: 30601830 PMC6314616

[ref128] Saigusa T. Dang Y. Bunni M. A. Amria M. Y. Steele S. L. Fitzgibbon W. R.. (2015). Activation of the intrarenal renin-angiotensin-system in murine polycystic kidney disease. Physiol. Rep. 3:e12405. 10.14814/phy2.12405, PMID: 25999403 PMC4463833

[ref129] Saigusa T. Dang Y. Mullick A. E. Yeh S. T. Zile M. R. Baicu C. F.. (2016). Suppressing angiotensinogen synthesis attenuates kidney cyst formation in a Pkd1 mouse model. FASEB J. 30, 370–379. 10.1096/fj.15-279299, PMID: 26391272 PMC4684522

[ref130] Salih M. Bovee D. M. Roksnoer L. C. W. Casteleijn N. F. Bakker S. J. L. Gansevoort R. T.. (2017). Urinary renin-angiotensin markers in polycystic kidney disease. Am. J. Physiol. Renal Physiol. 313, F874–F881. 10.1152/ajprenal.00209.2017, PMID: 28747358

[ref131] Santoso N. G. Cebotaru L. Guggino W. B. (2011). Polycystin-1, 2, and STIM1 interact with IP(3)R to modulate ER Ca release through the PI3K/Akt pathway. Cell. Physiol. Biochem. 27, 715–726. 10.1159/000330080, PMID: 21691089 PMC3221273

[ref132] Sherpa R. T. Mohieldin A. M. Pala R. Wachten D. Ostrom R. S. Nauli S. M. (2019). Sensory primary cilium is a responsive cAMP microdomain in renal epithelia. Sci. Rep. 9:6523. 10.1038/s41598-019-43002-2, PMID: 31024067 PMC6484033

[ref133] Soleimani A. Adabavazeh R. Nikoueinejad H. Sharif M. R. Faraji S. Otroshi Shahreza B.. (2015). Evaluation of Th17 pathway in the diagnosis of autosomal dominant polycystic kidney disease. Iran. J. Kidney Dis. 9, 105–112. PMID: 25851288

[ref134] Song X. Di Giovanni V. He N. Wang K. Ingram A. Rosenblum N. D.. (2009). Systems biology of autosomal dominant polycystic kidney disease (ADPKD): computational identification of gene expression pathways and integrated regulatory networks. Hum. Mol. Genet. 18, 2328–2343. 10.1093/hmg/ddp165, PMID: 19346236

[ref135] Spirli C. Locatelli L. Fiorotto R. Morell C. M. Fabris L. Pozzan T.. (2012). Altered store operated calcium entry increases cyclic 3′,5′-adenosine monophosphate production and extracellular signal-regulated kinases 1 and 2 phosphorylation in polycystin-2-defective cholangiocytes. Hepatology 55, 856–868. 10.1002/hep.24723, PMID: 21987453 PMC3272110

[ref136] Su Q. Hu F. Ge X. Lei J. Yu S. Wang T.. (2018). Structure of the human PKD1-PKD2 complex. Science 361:eaat981. 10.1126/science.aat9819, PMID: 30093605

[ref137] Sung P. H. Chiang H. J. Lee M. S. Chiang J. Y. Yip H. K. Yang Y. H. (2017). Combined renin-angiotensin-aldosterone system blockade and statin therapy effectively reduces the risk of cerebrovascular accident in autosomal dominant polycystic kidney disease: a nationwide population-based cohort study. Oncotarget 8, 61570–61582. 10.18632/oncotarget.18636, PMID: 28977886 PMC5617446

[ref138] Sweeney W. E. Frost P. Avner E. D. (2017). Tesevatinib ameliorates progression of polycystic kidney disease in rodent models of autosomal recessive polycystic kidney disease. World J. Nephrol. 6, 188–200. 10.5527/wjn.v6.i4.188, PMID: 28729967 PMC5500456

[ref139] Swenson-Fields K. I. Vivian C. J. Salah S. M. Peda J. D. Davis B. M. Van Rooijen N.. (2013). Macrophages promote polycystic kidney disease progression. Kidney Int. 83, 855–864. 10.1038/ki.2012.446, PMID: 23423256 PMC4028685

[ref140] Takenaka T. Kobori H. Inoue T. Miyazaki T. Suzuki H. Nishiyama A.. (2020). Klotho supplementation ameliorates blood pressure and renal function in DBA/2-pcy mice, a model of polycystic kidney disease. Am. J. Physiol. Renal Physiol. 318, F557–F564. 10.1152/ajprenal.00299.2019, PMID: 31928223

[ref141] Takiar V. Nishio S. Seo-Mayer P. King J. D. Jr. Li H. Zhang L.. (2011). Activating AMP-activated protein kinase (AMPK) slows renal cystogenesis. Proc. Natl. Acad. Sci. U. S. A. 108, 2462–2467. 10.1073/pnas.1011498108, PMID: 21262823 PMC3038735

[ref142] Tanner G. A. Tanner J. A. (2000). Citrate therapy for polycystic kidney disease in rats. Kidney Int. 58, 1859–1869. 10.1111/j.1523-1755.2000.00357.x, PMID: 11044205

[ref143] Taylor J. M. Hamilton-Reeves J. M. Sullivan D. K. Gibson C. A. Creed C. Carlson S. E.. (2017). Diet and polycystic kidney disease: A pilot intervention study. Clin. Nutr. 36, 458–466. 10.1016/j.clnu.2016.01.003, PMID: 26811129 PMC4940297

[ref144] Torres V. E. (2019). Pro: Tolvaptan delays the progression of autosomal dominant polycystic kidney disease. Nephrol. Dial. Transplant. 34, 30–34. 10.1093/ndt/gfy297, PMID: 30312438 PMC6657439

[ref145] Torres V. E. Abebe K. Z. Chapman A. B. Schrier R. W. Braun W. E. Steinman T. I.. (2014). Angiotensin blockade in late autosomal dominant polycystic kidney disease. N. Engl. J. Med. 371, 2267–2276. 10.1056/NEJMoa1402686, PMID: 25399731 PMC4284824

[ref146] Torres V. E. Abebe K. Z. Schrier R. W. Perrone R. D. Chapman A. B. Yu A. S.. (2017a). Dietary salt restriction is beneficial to the management of autosomal dominant polycystic kidney disease. Kidney Int. 91, 493–500. 10.1016/j.kint.2016.10.018, PMID: 27993381 PMC5237414

[ref147] Torres V. E. Chapman A. B. Devuyst O. Gansevoort R. T. Grantham J. J. Higashihara E.. (2012). Tolvaptan in patients with autosomal dominant polycystic kidney disease. N. Engl. J. Med. 367, 2407–2418. 10.1056/NEJMoa1205511, PMID: 23121377 PMC3760207

[ref148] Torres V. E. Chapman A. B. Devuyst O. Gansevoort R. T. Perrone R. D. Koch G.. (2017b). Tolvaptan in later-stage autosomal dominant polycystic kidney disease. N. Engl. J. Med. 377, 1930–1942. 10.1056/NEJMoa1710030, PMID: 29105594

[ref149] Torres V. E. Chapman A. B. Devuyst O. Gansevoort R. T. Perrone R. D. Lee J.. (2020). Multicenter study of long-term safety of tolvaptan in later-stage autosomal dominant polycystic kidney disease. Clin. J. Am. Soc. Nephrol. 16, 48–58. 10.2215/CJN.10250620, PMID: 33376102 PMC7792652

[ref150] Torres V. E. Grantham J. J. Chapman A. B. Mrug M. Bae K. T. King B. F. Jr.. (2011a). Potentially modifiable factors affecting the progression of autosomal dominant polycystic kidney disease. Clin. J. Am. Soc. Nephrol. 6, 640–647. 10.2215/CJN.03250410, PMID: 21088290 PMC3082424

[ref151] Torres V. E. Harris P. C. Pirson Y. (2007). Autosomal dominant polycystic kidney disease. Lancet 369, 1287–1301. 10.1016/S0140-6736(07)60601-1, PMID: 17434405

[ref152] Torres J. A. Kruger S. L. Broderick C. Amarlkhagva T. Agrawal S. Dodam J. R.. (2019). Ketosis ameliorates renal cyst growth in polycystic kidney disease. Cell Metab. 30, 1007.e1005–1023.e1005. 10.1016/j.cmet.2019.09.012, PMID: 31631001 PMC6904245

[ref153] Torres V. E. Meijer E. Bae K. T. Chapman A. B. Devuyst O. Gansevoort R. T.. (2011b). Rationale and design of the TEMPO (tolvaptan efficacy and safety in management of autosomal dominant polycystic kidney disease and its outcomes) 3-4 study. Am. J. Kidney Dis. 57, 692–699. 10.1053/j.ajkd.2010.11.029, PMID: 21333426

[ref154] Torres V. E. Ong A. C. M. (2020). Cellular signaling in PKD: foreword. Cell. Signal. 71:109625. 10.1016/j.cellsig.2020.109625, PMID: 32247773

[ref155] Verschuren E. H. J. Mohammed S. G. Leonhard W. N. Overmars-Bos C. Veraar K. Hoenderop J. G. J.. (2018). Polycystin-1 dysfunction impairs electrolyte and water handling in a renal precystic mouse model for ADPKD. Am. J. Physiol. Renal Physiol. 315, F537–F546. 10.1152/ajprenal.00622.2017, PMID: 29767557

[ref156] Walker R. V. Keynton J. L. Grimes D. T. Sreekumar V. Williams D. J. Esapa C.. (2019). Ciliary exclusion of Polycystin-2 promotes kidney cystogenesis in an autosomal dominant polycystic kidney disease model. Nat. Commun. 10:4072. 10.1038/s41467-019-12067-y, PMID: 31492868 PMC6731238

[ref157] Wang Q. Cobo-Stark P. Patel V. Somlo S. Han P. L. Igarashi P. (2018). Adenylyl cyclase 5 deficiency reduces renal cyclic AMP and cyst growth in an orthologous mouse model of polycystic kidney disease. Kidney Int. 93, 403–415. 10.1016/j.kint.2017.08.005, PMID: 29042084 PMC5794572

[ref158] Wang X. Constans M. M. Chebib F. T. Torres V. E. Pellegrini L. (2019a). Effect of a vasopressin V2 receptor antagonist on polycystic kidney disease development in a rat model. Am. J. Nephrol. 49, 487–493. 10.1159/000500667, PMID: 31117065 PMC6647848

[ref159] Wang X. Gattone V. 2nd Harris P. C. Torres V. E. (2005). Effectiveness of vasopressin V2 receptor antagonists OPC-31260 and OPC-41061 on polycystic kidney disease development in the PCK rat. J. Am. Soc. Nephrol. 16, 846–851. 10.1681/ASN.2004121090, PMID: 15728778

[ref160] Wang Z. Ng C. Liu X. Wang Y. Li B. Kashyap P.. (2019b). The ion channel function of polycystin-1 in the polycystin-1/polycystin-2 complex. EMBO Rep. 20:e48336. 10.15252/embr.201948336, PMID: 31441214 PMC6832002

[ref161] Warner G. Hein K. Z. Nin V. Edwards M. Chini C. C. Hopp K.. (2016). Food restriction ameliorates the development of polycystic kidney disease. J. Am. Soc. Nephrol. 27, 1437–1447. 10.1681/ASN.2015020132, PMID: 26538633 PMC4849816

[ref162] Watkins P. B. Lewis J. H. Kaplowitz N. Alpers D. H. Blais J. D. Smotzer D. M.. (2015). Clinical pattern of tolvaptan-associated liver injury in subjects with autosomal dominant polycystic kidney disease: analysis of clinical trials database. Drug Saf. 38, 1103–1113. 10.1007/s40264-015-0327-3, PMID: 26188764 PMC4608984

[ref163] Wong A. T. Y. Mannix C. Grantham J. J. Allman-Farinelli M. Badve S. V. Boudville N.. (2018). Randomised controlled trial to determine the efficacy and safety of prescribed water intake to prevent kidney failure due to autosomal dominant polycystic kidney disease (PREVENT-ADPKD). BMJ Open 8:e018794. 10.1136/bmjopen-2017-020068, PMID: 29358433 PMC5780847

[ref164] Woodward O. M. Watnick T. (2019). Molecular structure of the PKD protein complex finally solved. Am. J. Kidney Dis. 73, 620–623. 10.1053/j.ajkd.2018.12.022, PMID: 30704879

[ref165] Yacoub R. Nadkarni G. N. Mcskimming D. I. Chaves L. D. Abyad S. Bryniarski M. A.. (2019). Fecal microbiota analysis of polycystic kidney disease patients according to renal function: A pilot study. Exp. Biol. Med. (Maywood) 244, 505–513. 10.1177/1535370218818175, PMID: 30539656 PMC6547006

[ref166] Yook Y. J. Woo Y. M. Yang M. H. Ko J. Y. Kim B. H. Lee E. J.. (2012). Differential expression of PKD2-associated genes in autosomal dominant polycystic kidney disease. Genomics Inform. 10, 16–22. 10.5808/GI.2012.10.1.16, PMID: 23105924 PMC3475485

[ref167] Zhang Y. Dai Y. Raman A. Daniel E. Metcalf J. Reif G.. (2020). Overexpression of TGF-beta1 induces renal fibrosis and accelerates the decline in kidney function in polycystic kidney disease. Am. J. Physiol. Renal Physiol. 319, F1135–F1148. 10.1152/ajprenal.00366.2020, PMID: 33166182 PMC7792699

[ref168] Zhang B. Tran U. Wessely O. (2018). Polycystin 1 loss of function is directly linked to an imbalance in G-protein signaling in the kidney. Development 145:dev158931. 10.1242/dev.158352, PMID: 29530879 PMC5897598

[ref169] Zheleznova N. N. Wilson P. D. Staruschenko A. (2011). Epidermal growth factor-mediated proliferation and sodium transport in normal and PKD epithelial cells. Biochim. Biophys. Acta 1812, 1301–1313. 10.1016/j.bbadis.2010.10.004, PMID: 20959142 PMC3038174

[ref170] Zheng D. Wolfe M. Cowley B. D. Jr. Wallace D. P. Yamaguchi T. Grantham J. J. (2003). Urinary excretion of monocyte chemoattractant protein-1 in autosomal dominant polycystic kidney disease. J. Am. Soc. Nephrol. 14, 2588–2595. 10.1097/01.ASN.0000088720.61783.19, PMID: 14514736

[ref171] Zheng W. Yang X. Hu R. Cai R. Hofmann L. Wang Z.. (2019). Author correction: hydrophobic pore gates regulate ion permeation in polycystic kidney disease 2 and 2L1 channels. Nat. Commun. 10:1452. 10.1038/s41467-019-09422-4, PMID: 30914650 PMC6435729

[ref172] Zhou J. X. Fan L. X. Li X. Calvet J. P. Li X. (2015). TNFalpha signaling regulates cystic epithelial cell proliferation through Akt/mTOR and ERK/MAPK/Cdk2 mediated Id2 signaling. PLoS One 10:e0131043. 10.1371/journal.pone.0145421, PMID: 26110849 PMC4482222

[ref173] Zimmerman K. A. Bentley M. R. Lever J. M. Li Z. Crossman D. K. Song C. J.. (2019a). Single-cell RNA sequencing identifies candidate renal resident macrophage gene expression signatures across species. J. Am. Soc. Nephrol. 30, 767–781. 10.1681/ASN.2018090931, PMID: 30948627 PMC6493978

[ref174] Zimmerman K. A. Gonzalez N. M. Chumley P. Chacana T. Harrington L. E. Yoder B. K.. (2019b). Urinary T cells correlate with rate of renal function loss in autosomal dominant polycystic kidney disease. Physiol. Rep. 7:e13951. 10.14814/phy2.13951, PMID: 30632307 PMC6328912

[ref175] Zimmerman K. A. Hopp K. Mrug M. (2020). Role of chemokines, innate and adaptive immunity. Cell. Signal. 73:109647. 10.1016/j.cellsig.2020.109647, PMID: 32325183 PMC8063685

